# Transmissible H-aggregated NIR-II fluorophore to the tumor cell membrane for enhanced PTT and synergistic therapy of cancer

**DOI:** 10.1186/s40580-022-00352-4

**Published:** 2023-01-06

**Authors:** Haoli Yu, Yuesong Wang, Yan Chen, Mengyuan Cui, Fang Yang, Peng Wang, Min Ji

**Affiliations:** 1 State Key Laboratory of Bioelectronics, Jiangsu Laboratory for Biomaterials and Devices, School of Biological Science and Medical Engineering, Southeast University, Nanjing, 210096 China; 2 Department of Biomedical Engineering, School of Engineering, China Pharmaceutical University, Nanjing, 210009 China

**Keywords:** NIR-II fluorophore, H-aggregation, Tumor cell membrane, Liposome, PTT

## Abstract

Photothermal therapy (PTT) combined with second near-infrared (NIR-II) fluorescence imaging (FI) has received increasing attention owing to its capacity for precise diagnosis and real-time monitoring of the therapeutic effects. It is of great clinical value to study organic small molecular fluorophores with both PTT and NIR-II FI functions. In this work, we report a skillfully fluorescent lipid nanosystem, the RR_9_ (RGDRRRRRRRRRC) peptide-coated anionic liposome loaded with organic NIR-II fluorophore IR-1061 and chemotherapeutic drug carboplatin, which is named RRIALP-C4. According to the structural interaction between IR-1061 and phospholipid bilayer demonstrated by molecular dynamics simulations, IR-1061 is rationally designed to possess the H-aggregated state versus the free state, thus rendering RRIALP-C4 with the activated dual-channel integrated function of intravital NIR-II FI and NIR-I PTT. Functionalization of RRIALP-C4 with RR_9_ peptide endows the specifically targeting capacity for α_v_β_3_-overexpressed tumor cells and, more importantly, allows IR-1061 to transfer the H-aggregated state from liposomes to the tumor cell membrane through enhanced membrane fusion, thereby maintaining its PTT effect in tumor tissues. In vivo experiments demonstrate that RRIALP-C4 can effectively visualize tumor tissues and systemic blood vessels with a high sign-to-background ratio (SBR) to realize the synergistic treatment of thermochemotherapy by PTT synergistically with temperature-sensitive drug release. Therefore, the strategy of enhanced PTT through H-aggregation of NIR-II fluorophore in the tumor cell membrane has great potential for developing lipid nanosystems with integrated diagnosis and treatment function.

**Supplementary Information:**

The online version contains supplementary material available at 10.1186/s40580-022-00352-4.

## Introduction

Photothermal therapy (PTT) has been considered a benign therapy for cancer, which has the advantages of low trauma, significant therapeutic effects, and small side effects [1, 2]. In recent years, near-infrared (NIR) light-triggered PTT has held great promise for clinical tumor therapy owing to its deep tissue heating capability and has always cooperated with NIR fluorescence imaging (FI) to achieve the integration of diagnosis and treatment in tumor area [3, 4]. Moreover, FI in the NIR-II region (1000–1700 nm) possesses better spatial resolution and deeper tissue penetration due to lower tissue scattering and absorption [5–8]. Thus, current research has focused on NIR-II fluorescent materials with photothermal properties [9, 10].

The advantages of organic small molecular NIR-II fluorophores in biocompatibility and safety make them more suitable for clinical translation [11]. However, limited by the competitive relationship between radiative and nonradiative transitions, small organic molecular fluorophores often exhibit excellent fluorescence properties but lack efficient photothermal conversion efficiency [12, 13]. Even if the few kinds of literature realize the PTT of organic NIR-II fluorophores by twisted intramolecular charge transfer (TICT) with the help of nanocarriers such as albumin to forcibly change its original rigid structure, it will very well cause two potential defects: the destruction of its original NIR-II fluorescence and structural recovery after detachment from the nanomaterial in vivo [14–17]. Therefore, it is still crucial research to develop a new method for organic NIR-II fluorophores with efficient and stable photothermal properties for in vivo application.

Aggregation is a useful strategy to create a new set of features of small organic molecular fluorophores. Small organic molecular fluorophores usually possess two different aggregated states which are defined by the ways of their spatial arrangement [18]. In general, the spatial stacking mode of molecules dislocated stacking along the coplanar parallel direction is called the J-aggregated state, and overlapping and stacking face-to-face along the vertical direction of the coplanar is called the H-aggregated state [19]. Compared with the free state, molecules in aggregated states have different molecular energy levels, which will change their optical properties. Fluorophores in J-aggregated states tend to acquire the redshift and improved fluorescence spectra, which were beneficial for fluorescence imaging [20–22]. On the contrary, the H-aggregated state would change the electronic transition mode of fluorophores from radiation transition to non-radiation transition, decreasing the FI effect of fluorophores [23]. However, the H-aggregated state can convert the fluorescence properties of fluorophores into photothermal performance, which might be beneficial for improving the photothermal properties of NIR-II fluorescent molecules.

The aggregated state of fluorophores is usually influenced by the solvent environment and spatial distribution, which is determined by the materials in a nanosystem [15]. Moreover, most organic small molecular NIR-II fluorophores are suffered from extremely poor water solubility and often needed to combine with nanosystems [24]. Therefore, a rational design of a nanosystem can not only improve the biocompatibility of fluorophores but also possess an ideal spatial structure to change their aggregated state. In our previous research, we successfully constructed a novel NIR-II fluorescence liposomal platform that was able to change the aggregated state of fluorophores by rationally designing the steric hindrance and charge interaction between the bilayers of liposomes and fluorophores [25].

**Scheme 1:**
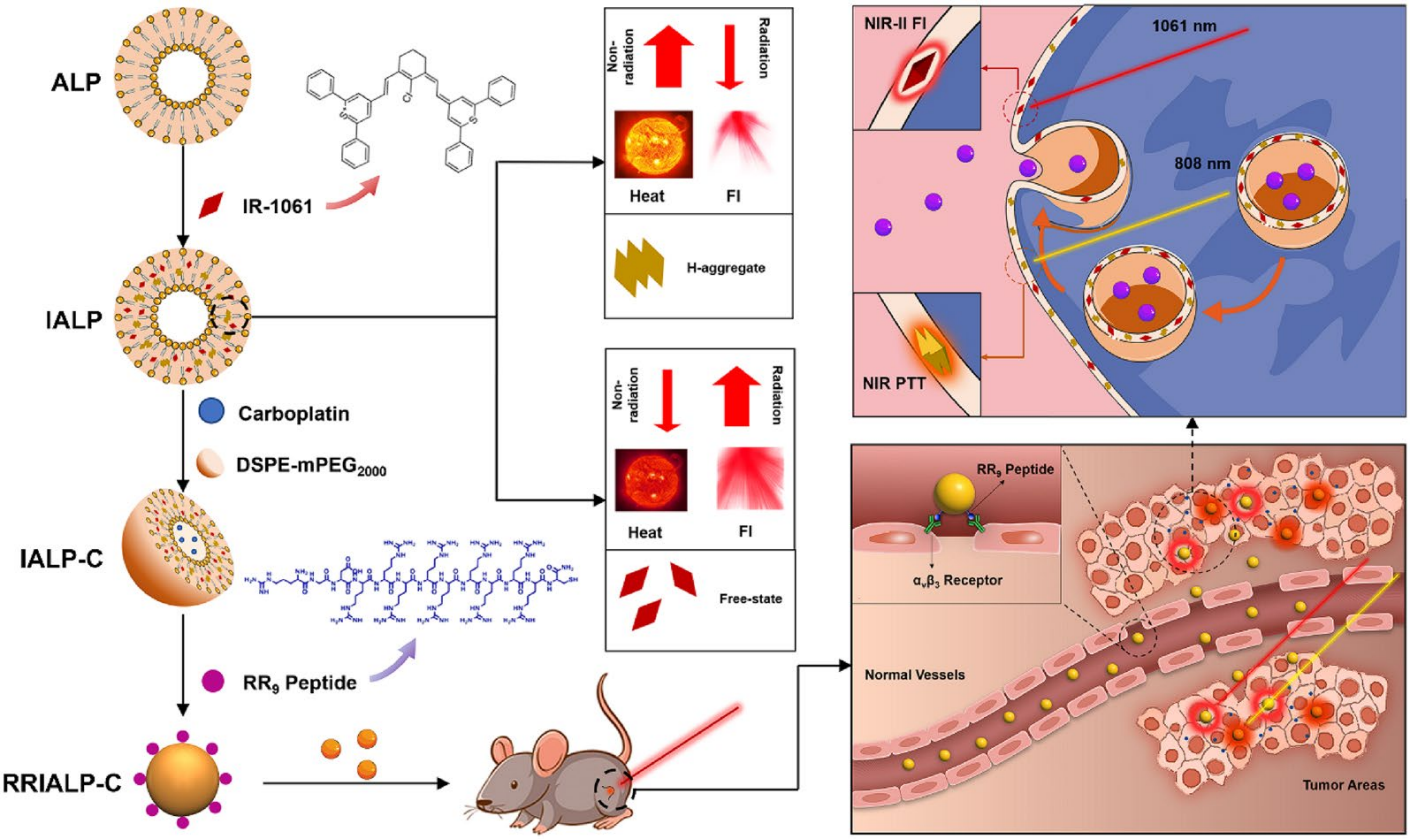
Schematic overview of experimental procedures. The rational design of RRIALP-C4 with the integration of intravital NIR-II FI and NIR-I PTT and the synergistic treatment of thermochemotherapy

In this work, we further visualized the interaction between phospholipid bilayer and NIR-II fluorophore IR-1061 through molecular dynamics simulation to clarify the way in which the H-aggregated state of IR-1061 formed in anionic liposomes (ALPs), and then proposed a method that may enable this aggregated state to be transmitted inside tumor cells to achieve stable and efficient PTT in vivo. On this foundation, a series of ALPs composed of negatively charged phospholipid DPPG, which contained both H-aggregated and free states of IR-1061, was prepared to realize NIR-II FI and PTT effect for lung cancer (Scheme 1). ALPs loaded IR-1061 (IALPs) possessed a low phase transition temperature at 41 °C which can provide thermoresponsive drug release capability when PTT occurs. Thus, when the chemotherapy drug carboplatin was encapsulated in IALPs (IALP-Cs), the synergistic treatment of thermochemotherapy in the tumor area was achieved. To enhance the tumor-targeting ability of IALP-Cs, we systematically investigated the factors that affect liposomes uptake by A549 cells through modification of ALPs with three different peptides, RGD (Arg-Gly-Asp), TAT_48–60_ (GRKKRRQRRRPPQ) and RR_9_ (RGDRRRRRRRRRC). RGD peptide can be specifically targeted α_v_β_3_ highly expressed endothelial cells of tumor vasculature and TAT_48–60_ peptide is known as a cell membrane penetrating peptide because of its large number of positive charges [26–28]. RR_9_ peptide is synthesized from a stretch of positively charged arginine chains with RGD peptide, which integrates the functions of both peptides [29]. In addition, the uptake mechanism of ALPs and the PTT effect after uptaken by A549 cells were also evaluated to investigate whether the intraliposomal H-aggregated state of IR-1061 could be maintained inside tumor cells. Importantly, the optimized IALP, RR_9_-coated IALP-C4 (RRIALP-C4), was successfully applied in the synergistic thermochemotherapy under the guidance of NIR-II FI and NIR-I photothermal imaging (PTI) both in vitro and in vivo. This work innovatively develops a fluorescence liposome system with simultaneous aggregated and free states of IR-1061, providing a promising dual-channel activated integrated function for the diagnosis and treatment of tumors.

## Results and discussions

### Synthesis and characterization of IALPs

**Fig. 1:**
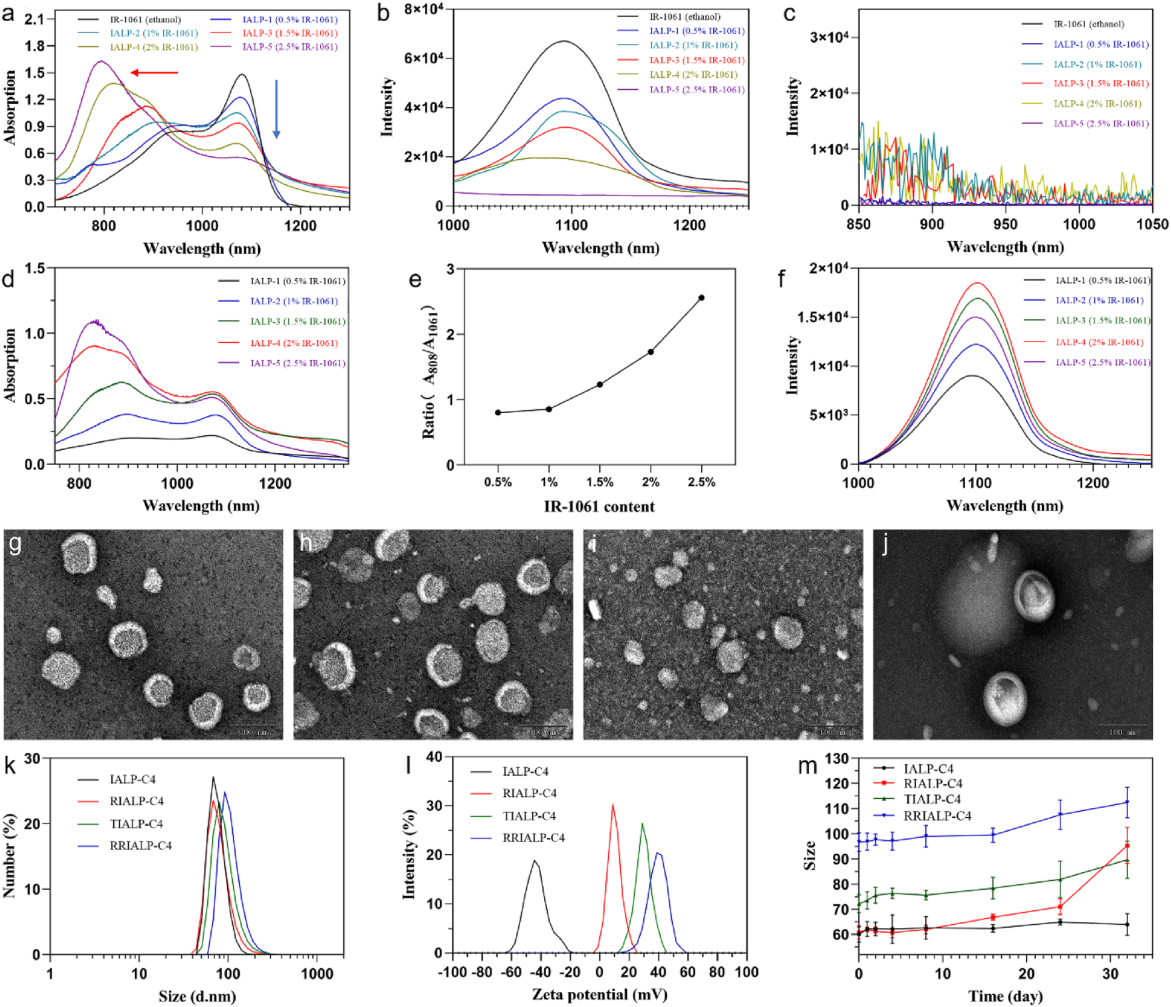
Characterization of liposomes. **a** Absorption spectra of ALPs with different molar contents of IR-1061 (same concentrations of IR-1061). **b**, **c** Fluorescence spectra ALPs with different IR-1061 contents (**b** 1061 nm excitation; **c** 808 nm excitation). **d** Absorption spectra of ALPs at the same concentrations of phospholipid. **e** A_808_/A_1061_ ratio of ALPs with different IR-1061 contents. **f** Fluorescence spectra of ALPs at 1061 nm excitation. **g**–**j** TEM images of IALP-C4, RIALP-C4, TIALP-C4 and RRIALP-C4. **k**, **l** Hydrodynamic size and zeta potentials of IALP-C4, RIALP-C4, TIALP-C4, and RRIALP-C4. **m** Size changes of liposomes placed at 4 °C for 30 days

To investigate the occurrence of the H-aggregated state of IR-1061 in ALPs and its mechanism, a total of 5 kinds of ALPs, named IALP-1 (2, 3, 4, 5), with different molar contents (compared with phospholipid) of IR-1061 (0.5, 1, 1.5, 2, and 2.5%) were synthesized and their optical spectral properties were analyzed. As shown in Fig. 1a, at the same solution concentration of IR-1061, the main absorption peak at 1061 nm gradually decreased with the increase of molar contents of IR-1061 in ALPs. On the contrary, another absorption peak appeared with continuous blue shift and intensity enhancement. The fluorescence spectra of IALPs showed that the main fluorescence emission peak at 1100 nm decreased corresponding to the absorption spectrum under the excitation of 1061 nm laser. There is no new shorter wavelength emission peak appeared under the excitation of 808 nm laser, demonstrating that the new absorption did not release energy in the form of fluorescence (Fig. 1b, c). The characteristics of the blue shift peak are completely consistent with that of the fluorophore under the H-aggregated state, meaning the increase of IR-1061 content in IALPs will increase its proportion in the H-aggregated state and reduce that of the free state [30]. Furthermore, the absorption spectra of IALPs at the same solution concentration of phospholipid showed that the absorbance of IR-1061 at 1061 nm increased at low molar contents, no longer increased over 1.5% molar content, and even began to decrease at 2.5% molar content (Fig. 1d). Whereas the absorbance at 890 nm increased more rapidly with the increase of IR-1061 content (Fig. 1e). The cessation of the increased absorption at 1061 nm demonstrated that the free state of IR-1061 could reach the maximum when the molar content of IR-1061 in IALPs is over 1.5%, and then the excess fluorophore might completely exist in the H-aggregated state. The results of fluorescence intensity of IR-1061 at 1061 nm with different concentrations were under our discussion (Fig. 1f).

In terms of the above experimental results, the anionic liposome with 1.5% IR-1061 (IALP-3) and 2% IR-1061 (IALP-4) will be considered as the optimal fluorescence liposomes. On the other hand, the higher the molar content of IR-1061 with an H-aggregated state exists in liposomes, the more excellent its photothermal performance presents. Thus, we believe that IALP-4 is more suitable for simultaneous PTT and NIR-II FI. Next, liposomes modified with three types of peptides (RGD, TAT_48–60_, and RR_9_) and encapsulated with carboplatin were named RIALP-C4, TIALP-C4, and RRIALP-C4, were for subsequent studies. Both absorption and fluorescence spectra of these liposomes illustrated that the modification of peptides and the encapsulation of carboplatin do not affect their optical properties (Additional file 1: Fig. S1). As shown in Fig. 1k, the mean hydrodynamic size of the IALP-C4, RIALP-C4, TIALP-C4, and RRIALP-C4 was 51 nm (with polymer dispersity index, PDI: 0.138), 48 nm (PDI: 0.154), 61 nm (PDI: 0.135), and 95 nm (PDI: 0.165), respectively, meaning that the modification of peptides will slightly increase the particle size of liposomes but not affect the uniform dispersion. TEM results presented the homogeneous distributed roughly spherical particles with liposomal characteristic bimolecular layer structure (Fig. 1g–j). The ζ potential confirmed the successful modification of different peptides as well as the change of liposome surface charges (Fig. 1L). Figure 1m showed the size changes of different modified types of IALP-C4 at 4 °C for 1 month. The results demonstrated that IALP-C4 possessed the best stability due to its negative charge on its surface, while RIALP-C4 possessed the worst stability because there is not enough positive charge on its surface. TIALP-C4 and RRIALP-C4 showed almost no size changes within 3 weeks and slight size changes within 1 month, indicating the positive charge modifications of IALP-C4 also provided excellent stability.

### Evaluation of the molecular dynamics simulations of IALPs

**Fig. 2:**
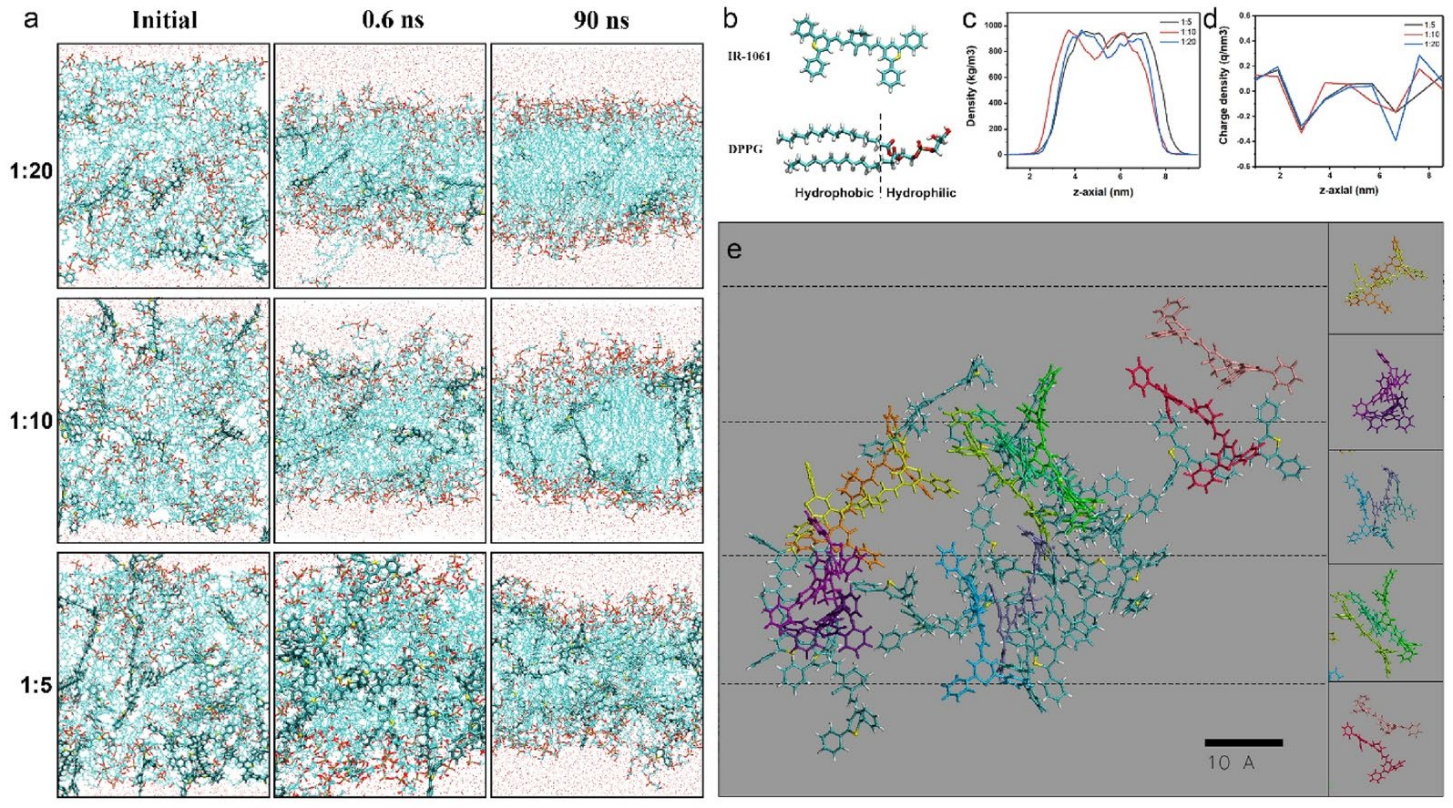
Molecular dynamics simulation of IR-1061 in DPPG. **a** Operation procedure of systems with different number ratios of IR-1061 and phospholipid. **b** Molecular structure of IR-1061 and DPPG. **c**, **d** Mass density distribution and charge density distribution of systems after running for 90 ns. **e** The H-aggregated state of IR-1061 in the system (the number ratio of IR-1061 to DPPG is 1: 5) after running for 90 ns (dash line: distribution layers of IR-1061 in H-aggregated state)

To better understand the effect of phospholipid bilayer on the structure and spectral properties of IR-1061, we performed molecular dynamics simulations of IR-1061 with different concentrations in the phospholipid bilayer. In this work, three different number ratios of IR-1061 and DPPG molecules (1: 20, 1: 10, and 1: 5) were used to construct the bilayers to simplify the calculation (Fig. 2b). A total of 120 phospholipid molecules with a corresponding ratio of IR-1061 molecules were put into the system and operated for 90 ns. The operation process was shown in Fig. 2a. After placing the chaotic system in the cube in the initial setup, the hydrophobic and hydrophilic groups of DPPG began to separate within 0.6 ns and reached a plateau after 90 ns, showing the appearance of a classical phospholipid bilayer structure. Compared with the system with high IR-1061 concentration, the system with low IR-1061 concentration showed a smaller phospholipid bilayer gap and more uniform hydrophilic and hydrophobic layer separation. These results were also demonstrated in Fig. 2c, d. The mass density distribution of systems showed that the total thickness of the liposome bilayer was about 6 nm, and the thickness of the hydrophobic layer ranged from 3 to 3.5 nm. Among them, after the number of IR-1061 increased, the hydrophobic bilayer broadened and the central density increased. The charge density distribution of systems showed that the high concentration of IR-1061 increased the distribution space of the negative charge of DPPG, which proved that the increase of IR-1061 would interfere with the uniformity of the phospholipid hydrophilic layer.

Subsequently, the number and the proportion of IR-1061 in the H-aggregated state were evaluated in the molecular dynamics simulation results. The evaluation criteria of IR-1061 in the H-aggregated state is that 2 molecules are arranged face-to-face with a distance less than 5 Å. In the system with a 1: 5 ratio of IR-1061 to DPPG, 12 IR-1601 molecules were in the H-aggregated state after 90 ns of operation, accounting for 50% of the total number of IR-1061 molecules (Fig. 2e). In the other two systems, the proportion of IR-1061 in the aggregated state is 0% and 33% respectively (Additional file 1: Fig. S2). The results were consistent with our experimental results and made us observe the cause of this phenomenon more intuitively. The distribution space of the phospholipid bilayer enables only one IR-1061 molecule vertically distributed. Therefore, the vertical distribution space of IR-1061 is greatly limited, in turn forming a horizontal, face-to-face distribution. When the number of IR-1061 increased, the number of horizontally distributed IR-1061 layers also increased (shown in the dashed lines in Fig. 2e), and the molecular gaps and misplacement angles of IR-1061 became smaller and smaller, which eventually formed the H-aggregated state.

### Evaluation of the photothermal performance of RRIAP-C4

**Fig. 3:**
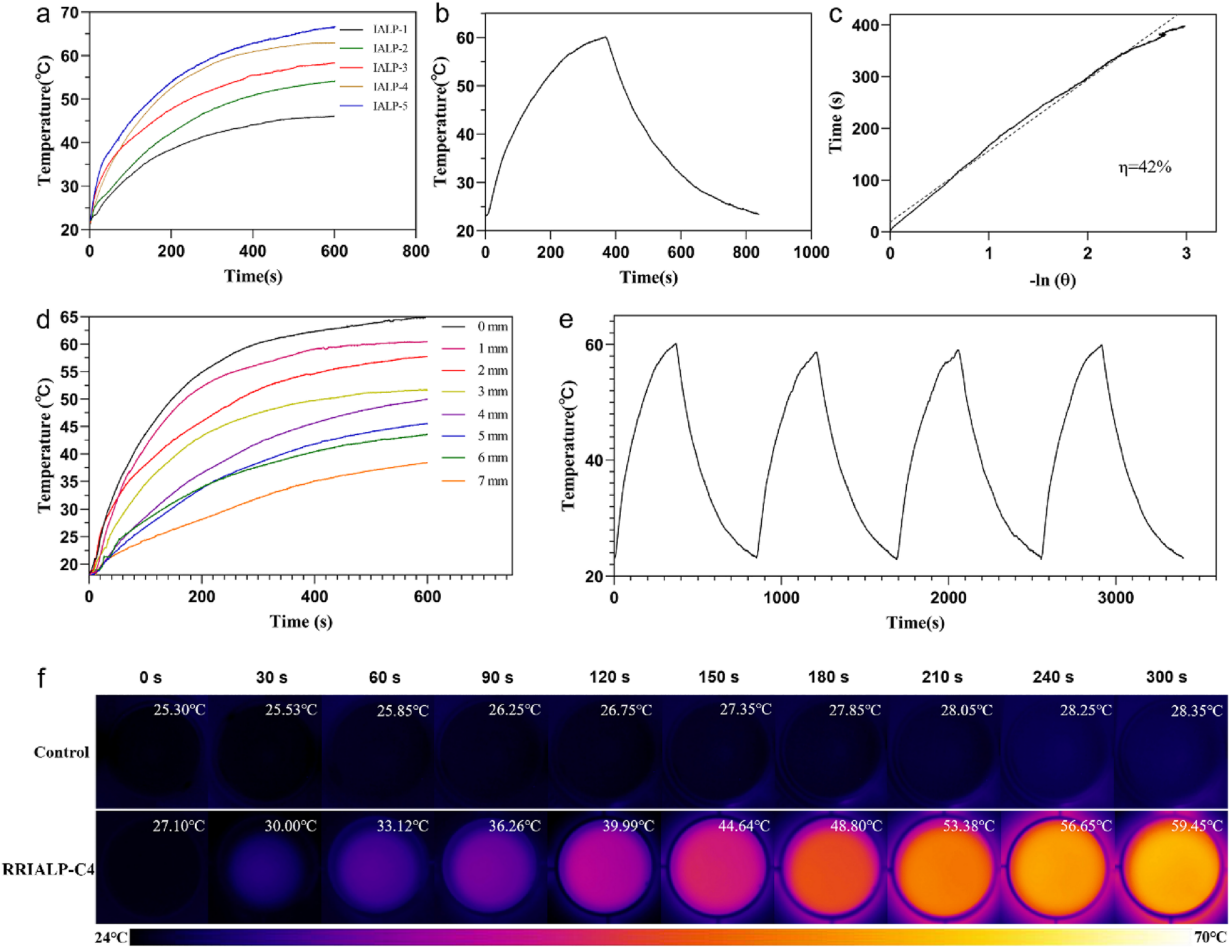
Photothermal performance of liposomes. **a** Temperature changes of IALPs (phospholipid concentration: 10 mg/mL) after being irradiated with 808 nm laser at 0.3 W/cm^2^. **b** Temperature changes of RRIALP-C4 (10 mg/mL) irradiated by 808 nm laser at 0.3 W/cm^2^ and cooled at room temperature. **c** The photothermal conversion efficiency RRIALP-C4 (10 mg/mL) was calculated from the linear fitting of − ln^θ^ and time in the cooling curve. **d** Temperature changes of RRIALP-C4 (10 mg/mL) coated with different thicknesses of chicken tissues after being irradiated with 808 nm laser at 0.3 W/cm^2^. **e** Photothermal stability of RRIALP-C4 (laser on/off for 4 consecutive cycles) irradiated with 808 nm laser at 0.3 W/cm^2^. **f** Thermal images of RRIALP-C4 (10 mg/mL) after being irradiated with 808 nm laser at 0.3 W/cm^2^ in 24-well plates (control: cell culture medium)

The H-aggregated state of fluorophores will convert the light absorbed from a radiative state into a non-radiative state, which will induce the photothermal conversion [31]. Therefore, the photothermal properties of IALPs were examined by thermometry and thermal imaging. First, the heating curves of IALPs with different molar contents of IR-1061 were tested (Fig. 3a). The results showed that the heating capacity of IALPs was positively correlated with the molar content of IR-1061, which verified our previous conjecture. Next, more photothermal properties of the selected liposome RRILAP-C4 were detected to evaluate its PTT effect. As shown in Fig. 3b, c, the cooling curve and t − (− ln^θ^) plot of RRILAP-C4 were made to show a stable photothermal conversion efficiency (the R^2^ value of the straight-line fit was more than 0.99). The photothermal conversion efficiency of RRILAP-C4 was calculated to be 42% according to the curves, which was just a sight lower than RRIALP-C5, but much higher than another NIR fluorophore, IR-780 (Additional file 1: Figs. S3a−d, S4a). The photostability of RRIALP-C4 was also examined by cyclic lift temperature (Fig. 3e). Moreover, to prove the excellent photothermal performance of RRIALP-C4, we simultaneously prepared ICG-encapsulated ALP (ICG-ALP) and IR-780-loaded ALP (IR780-ALP), and tested their photothermal stability (Additional file 1: Figs. S3e, S4b). ICG is a clinical contrast agent and has been proved to possess photothermal properties. IR-780 was also reported to have good photothermal properties [32]. The results showed that RRIALP-C4 had a higher rising temperature and more cycle times, demonstrating that RRIALP-C4 possessed excellent photothermal property and stability far beyond ICG-ALP. Moreover, to test the IR-1061-loading stability of RRIALP-C4, we circulated the temperature of RRIALP-C4 for 0, 1, 2, 3, 4 times respectively, and measured its absorption after the liposome is dialyzed (Additional file 1: Fig. S5). The results showed extremely low leakage of IR-1061 and unreduced H-aggregated form under short-term temperature circulations.

Furthermore, to test the heating effect of RRIALP-C4 in cell experiments, RRIALP-C4 solutions with different concentrations (diluted by cell culture medium) were placed in 24-well plates and their temperature rise was monitored by the thermal imager. The pictures in Fig. 3f displayed that the temperature of RRIAP-C4 solution at 10 mg/mL increased from 27 to 59 °C within 300 s, which was significantly higher than that of the control group. Moreover, the different concentrations of RRIALP-C4 were also evaluated (Additional file 1: Fig. S6). The temperature could rise to the effective treatment temperature (over 42 °C) in a short time even when the phospholipid concentration of RRIALP-C4 was low to 1 mg/mL, indicating RRIALP-C4 have an effective PTT effect on tumor cells.

The simulation experiment of tissue photothermal penetration depth was also carried out using chicken breast tissues in vitro. As shown in Fig. 3d, the maximum temperature of the RRIALP-C4 solution displayed a stepwise decrease with the increase of chicken tissue thickness. The maximum chicken tissue thickness to reach effective treatment temperature was 6 mm, and that to realize rapid heating was 4 mm, indicating RRIALP-C4 has a good tissue penetration depth for tumor PTT.

### In vitro synergistic therapy and target delivery

**Fig. 4:**
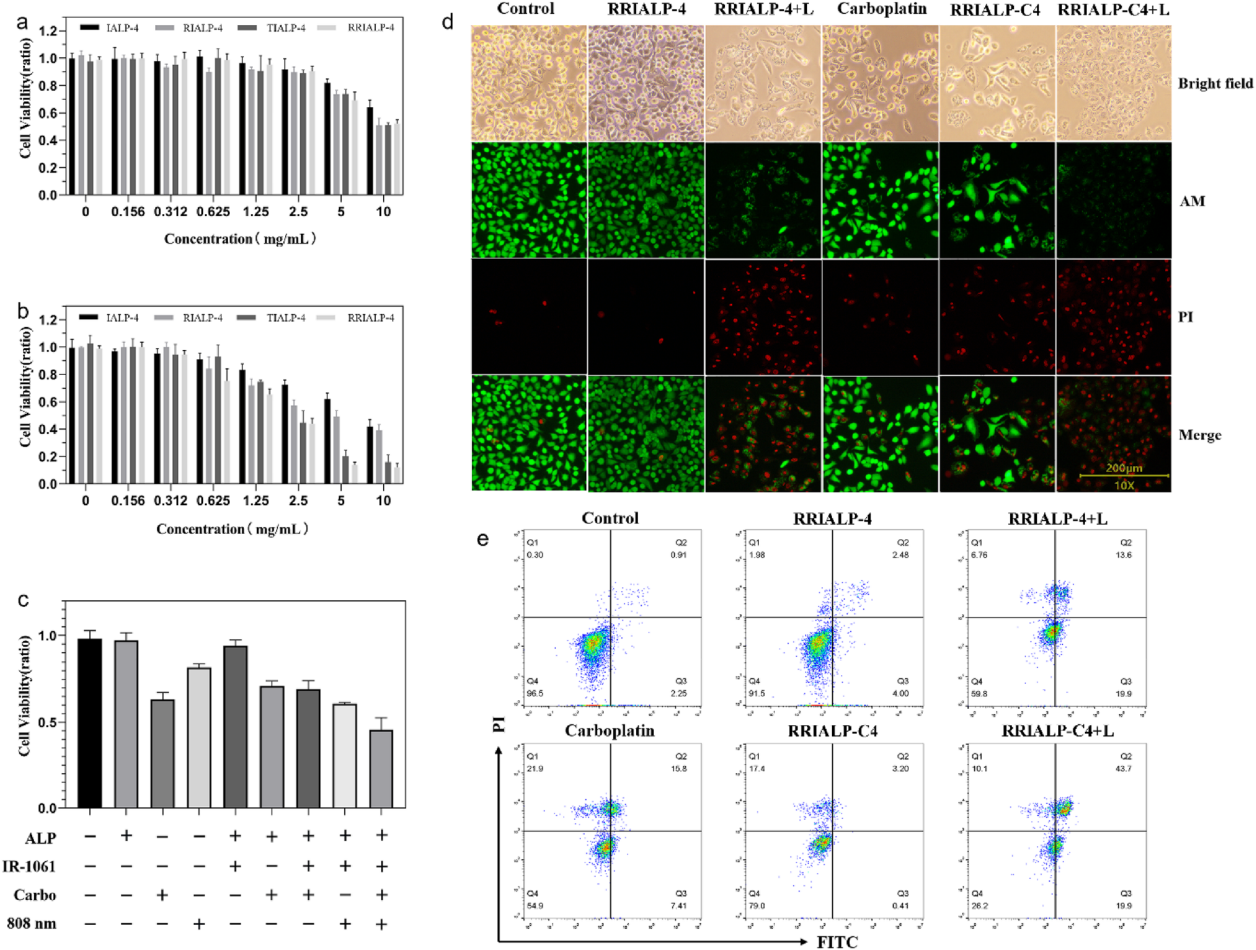
In vitro photothermal therapy and synergistic treatment for A549 cells. **a** Cytotoxicity of different phospholipid concentrations of ALPs in A549 cells after treatment for 24 h. **b** Cytotoxicity of A549 cells treated with different phospholipid concentrations of ALPs for 30 min and irradiated with 808 nm laser at 0.3 W/cm^2^ for 6 min. **c** Cell viability of A549 cells treated with liposomes at different conditions (Liposomes: 1 mg/mL; Carbo: 6.7 µM). **d** Fluorescent staining of dead/living cells treated with different conditions (L represented irradiation with 808 nm laser at 0.3 W/cm^2^). Green channel: viable cells. Red channel: dead cells. **e** Flow cytometry of apoptosis cells treated with different conditions. Q1, Q2, Q3, and Q4 represent necrotic, late apoptotic, early apoptotic, and viable cells, respectively. (Liposomes: 1 mg/mL; Carbo: 6.7 µM)

The cytotoxicity of IALPs with different phospholipid concentrations to A549 cells was evaluated by cell viability assay to evaluate their biocompatibility. As shown in Fig. 4a, RRIALP-4 had little significant cytotoxicity (> 95%) to A549 cells at a phospholipid concentration lower than 2 mg/mL, which contained the appropriate concentration for tumor PTT. The results of RIALP-4 and TIALP-4 were consistent with RRIALP-4, indicating that the modification of different types of peptides had no obvious effect on the toxicity of IALPs. To test the influence of modification of different peptides on the PTT of IALP-4s, A549 cells were incubated with IALP-4s for 30 min, and then the samples were replaced by the new culture medium. After the irradiation of 808 nm laser (0.3 W/cm^2^) for another 6 min, all IALPs showed a certain degree of toxicity (Fig. 4b). Compared with IALP-C4 and RIALP-C4, TIALP-C4 and RRIALP-C4 showed more damage to A549 cells under irradiation. Especially, RRIALP-C4 possessed significant cytotoxicity even at the concentration of 0.625 mg/mL (< 80%) and could reach the semi-inhibitory concentration at 2.5 mg/mL, indicating that the suitable concentration of RRIALP-C4 for PTT is about 2 mg/mL, which will offer not only effective PTT effect but also good biosafety. The results of significant differences in cytotoxicity between IALP-C4s demonstrated that the modification of different peptides on IALP-4s significantly affected their PTT effect in vitro, which might be related to the accumulation of liposomes in tumor cells. ICG aqueous solution and ICG-loaded ALP (2% molar content) were also detected as control groups (Additional file 1: Fig. S7). The results showed ICG-ALP also possessed excellent photothermal treatment effect.

The synergistic therapy of RRIALPs (1 mg/mL) to A549 cells was detected by cell viability detection and cell apoptosis detection. The effects of liposomes under different conditions on cell activity were shown in Fig. 4c. There was no significant cytotoxicity when blank RRALP (cell viability: 97.5% ± 1.8%) and RRIALP-4 (95.3 ± 2.6%) interacted with A549 cells, while the carboplatin-loaded RRALPs possessed certain cytotoxicity (RRALP-C: 73.5% ± 1.4%, RRIALP-C4: 72.1% ± 3.2%). The irradiation of 808 nm laser showed slight damage on A549 cells (86.4% ± 0.91%), but much lower than that co-operated with RRIALP-4 (65.2% ± 0.33%). More importantly, the greatest cytotoxicity (44.7% ± 6.1%) was exhibited when carboplatin synergized with the liposomal photothermal effect, indicating that the synergistic effect of thermochemotherapy was better than each one. Fluorescence signals of live/dead cells by fluorescence microscope showed the same trend as cell activity that the group of RRIALP-C4 with irradiation possessed the lowest fluorescence of living cells and the highest fluorescence of dead cells (Fig. 4d). Furthermore, cell apoptosis was quantitatively analyzed by flow cytometry (Fig. 4e). The carboplatin treatment groups presented more injured cells concentrated in the cell death region (Q1), while most of the injured cells in the PTT groups belonged to cell apoptotic region (Q2 and Q3). The results of cell death mode in these two ways correspond to their mechanism. It is known that carboplatin drugs directly act on DNA in the nucleus to cause cell damage from inside to outside, therefore apoptotic staining exhibits high PI signals and low FITC signals [33]. On the contrary, thermotherapy exerts its cytotoxic effect mainly by inducing apoptosis at 42–45 °C, thus exhibiting normal apoptosis staining results [34]. Whereas, the proportion of apoptosis was greatly promoted in the group of RRIALP-C4 with irradiation compared with the group of RRIALP-C4 alone, demonstrating the synergistic therapy of carboplatin and photothermal both played a role.

The combinations of drugs (free carboplatin + IR-1061-loaded ALP) and single drug-loaded liposomes (carboplatin-loaded liposome + IR-1061-loaded ALP) were also supplemented to examine the combinational index of carboplatin and IR-1061 (Additional file 1: Fig. S8). The free drugs group exist the highest cell lethality (60%), which was nearly the sum of single drugs. The single liposomes combi group showed a cell lethality (42%), which was lower than IALP-C4 and free drugs group. The combinational index of Carbo + IALP-4, ALP-C + IALP-4, and IALP-C4 were calculated respectively. The combinational index of carboplatin and IALP-4 is 0.85, which means a low synergy effect. The combinational index of IALP-C4 is 0.95, which means an additive effect. The combinational index of ALP-C and IALP-4 is higher than 1, which means an antagonistic effect. We thought the reason for the difference of free drugs group, single liposomes combi group, and IALP-C4 group is that the uptake of liposome by A549 cell is limited.

**Fig. 5:**
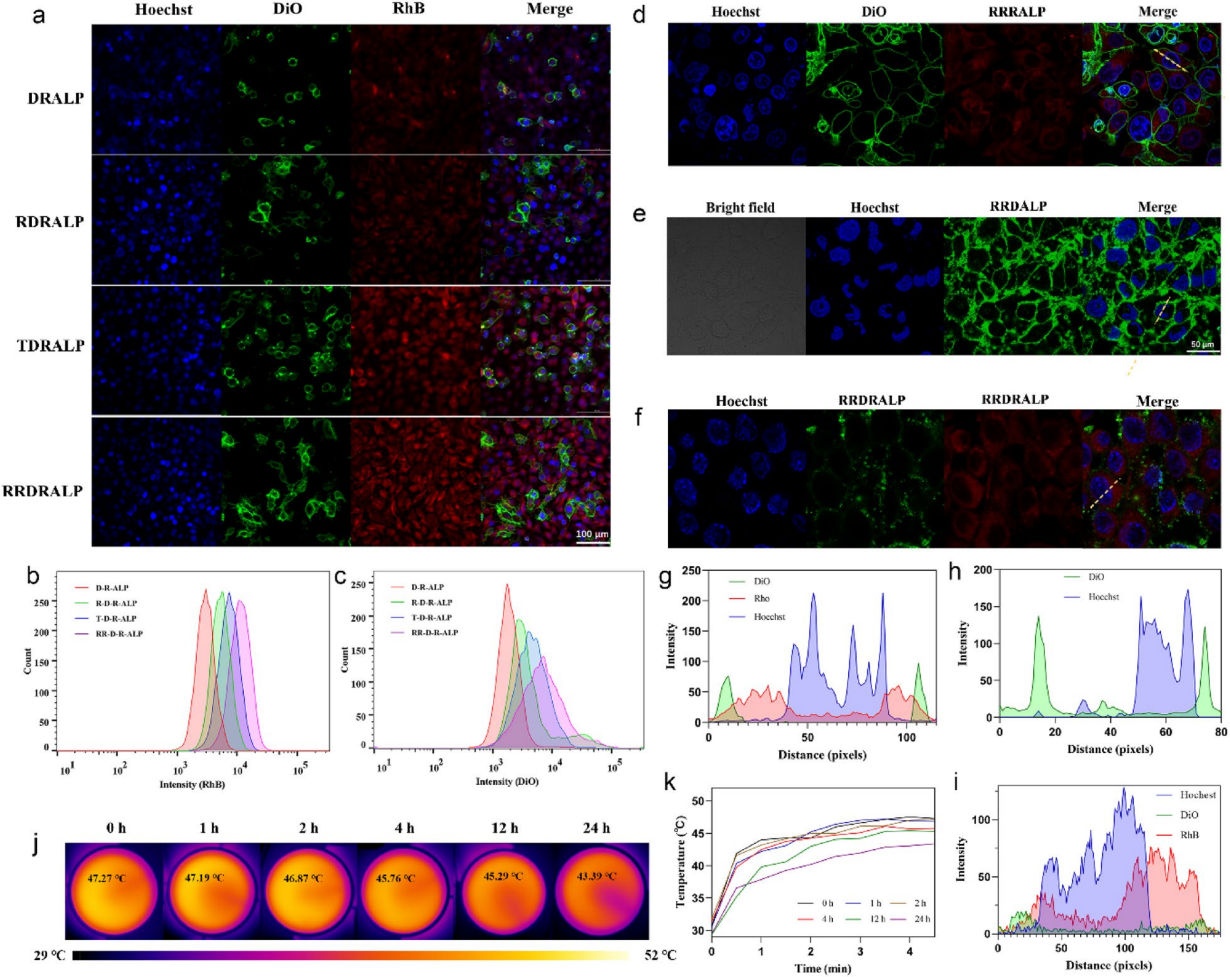
Cell targeting and uptakes of liposomes. **a** CLSM images of A549 cells treated with DRALPs for 30 min (Blue channel: nucleus; Green and Red channels: liposomes). **b**, **c** Fluorescence analysis of RhB and DiO in A549 cells. **d**–**f** CLSM images (100× oil) of A549 cells treated with 2 mg/mL RRRALP, RRDALP, and RRDRALP respectively (the stain of the membrane in the RRRALP group was DiO alone). **g**–**i** Fluorescence profile analysis of the yellow dashes in CLSM images. **j**, **k** Thermal images of A549 cells treated with RRIALP-4 (2 mg/mL) in different times (irradiated with 808 nm laser at 0.3 W/cm^2^ for 5 min)

The target delivery of ALPs with the modification of different types of peptides was evaluated through laser confocal microscopy imaging (CLSM). Since IR-1061 is a NIR-II fluorophore, DiO and Rhodamine B (RhB), two fluorophores in the visible region, were replaced in the ALPs (DRALPs) to realize the cellular visualization in further experiments. The fluorescence properties of these two dyes in ALP were detected to ensure the reliability of fluorescent signals (Additional file 1: Fig. S10). As shown in Fig. 5a, the successful FI of DiO and RhB in A549 cells proved that DRALPs did enter the cells in varying degrees. Compared with unmodified DRALPs, more amount of peptide-modified DRALPs had entered A549 cells. More accurate fluorescence intensity was analyzed through flow cytometry (Fig. 5b, c). Interestingly, Compared with RGD-modified DRALP (RDRALP), we found that A549 cells uptake more TAT-modified DRALP (TDRALP), demonstrating that the charge modification on the surface of ALPs is more conducive to liposome entry into A549 cells than receptor targeting. By integrating RGD peptide function and TAT peptide function, RR_9_ modified DRALP (RRDRALP) had the maximum fluorescence intensity. It proved that the synergy of the two functional peptides maximized the tumor-targeting effect of ALPs. To further prove the way of liposomes entering A549 cells, we used the endocytosis inhibitor, Pitstop 2, to inhibit the clathrin mediated cytocytosis (Additional file 1: Fig. S13). The results showed that Pitstop 2 cannot prevent RRDRALP into A549 cells, which means RRDRALP does not enter the cell through the way of endocytosis.

We have achieved the coexistence of the H-aggregated state of IR-1061 with the free state through the unique phospholipid bilayer structure of liposomes, which cannot be achieved in other solvent environments. Therefore, whether liposomes still maintain their dual states after uptake by tumor cells, especially the H-aggregated state, is need to be investigated. We visualized the distribution as well as the status of fluorophores in liposomes during phagocytosis by investigating the tumor cell uptake mechanism of ALPs. Liposomes generally enter cells through cell membrane fusion and endocytosis, each of which can be enhanced in different ways [35, 36]. RhB is a water-soluble fluorophore, which can only prove that liposomes do enter cells but cannot distinguish the way liposomes enter cells. While DiO is a hydrophobic fluorophore that it can further determine the way liposomes enter cells through its cellular distribution. For this reason, RhB-ALPs (RALPs) and DiO-ALPs (DALPs) were prepared separately and collocated their confocal fluorescence signals with DRALPs to evaluate the uptake mechanism of ALPs. As shown in Fig. 5d, g, the confocal through the 100× oil lens of the confocal microscope, the fluorescence of the nucleus, RhB, and cell membrane (stained by DiO dye alone) showed a significant distinction boundary, indicating that RhB was distributed in the cytoplasm. The modification of liposome surface peptide only changed the amount of RhB in cells but did not change its distribution (Additional file 1: Fig. S9a). While in the RRDALP group, the vast majority of DiO showed a distribution around the cells (Fig. 5e). Compared with RhB, there was a significant gap between the fluorescence signals of DiO and the nucleus, demonstrating that DiO was still abundantly present on the cell membrane (Fig. 5h). Only a very small amount of DiO fluorescence signal appeared in the cells for the RR_9_-modified DAP (RRDALP) and even less for the other peptide-modified DALPs, which proved that ALPs entered A549 cells mainly through membrane fusion (Additional file 1: Fig. S9b). In the RRDRALP group, even if DIO and RhB were in the same liposome, the distribution of these two dyes was completely consistent with that of the first two groups (Fig. 5f, i).

These results proved that IALPs were absorbed by A549 cells in the form of membrane fusion, and the distribution of IR-1061 would be dispersed on the cell membrane, similar with DiO. This distribution would make it easier for IR-1061 to maintain its H-aggregated state and still possess the PTT effect, which was also confirmed by other experiments. The A549 cells incubated with RRIALP-4 at different times, and then detected their temperature rise curves with an infrared thermal imager. As shown in Fig. 5j, k, and Additional file 1: Fig. S11, RRIALP-4 could maintain an effective PTT effect when co-incubation with A549 cells for more than 24 h. The decrease of maximum temperature after 12 h might be the fluidity of the cell membrane dispersing part of IR-1061 in the aggregated state. Another experiment was carried to stronger this evidence. We detected the absorption of purified cell membrane after incubated with IALP-4 (Additional file 1: Fig. S18). Specifically, after incubating A549 cells with IALP-4 for different time, we removed free liposomes by centrifugation, then cells were split using cell lysate and freeze-thaw method. The organelles are removed again by centrifugation to obtain cell membranes. The absorption peaks of cell membrane showed an obvious characteristic peak of IR-1061 in H-aggregated state at 800 nm, indicating that IR-1061 exists in the form of H-aggregates on the cell membrane.

ICG aqueous solution and ICG-loaded ALP (2% molar content) were also detected as control groups (Additional file 1: Fig. S12). The results showed both groups could only maintain their photothermal treatment effect in 2 h. This is due to their poor photothermal stability, and they cannot repeat photothermal cycles.

### NIR-II FI properties of RRIALP-C4

**Fig. 6:**
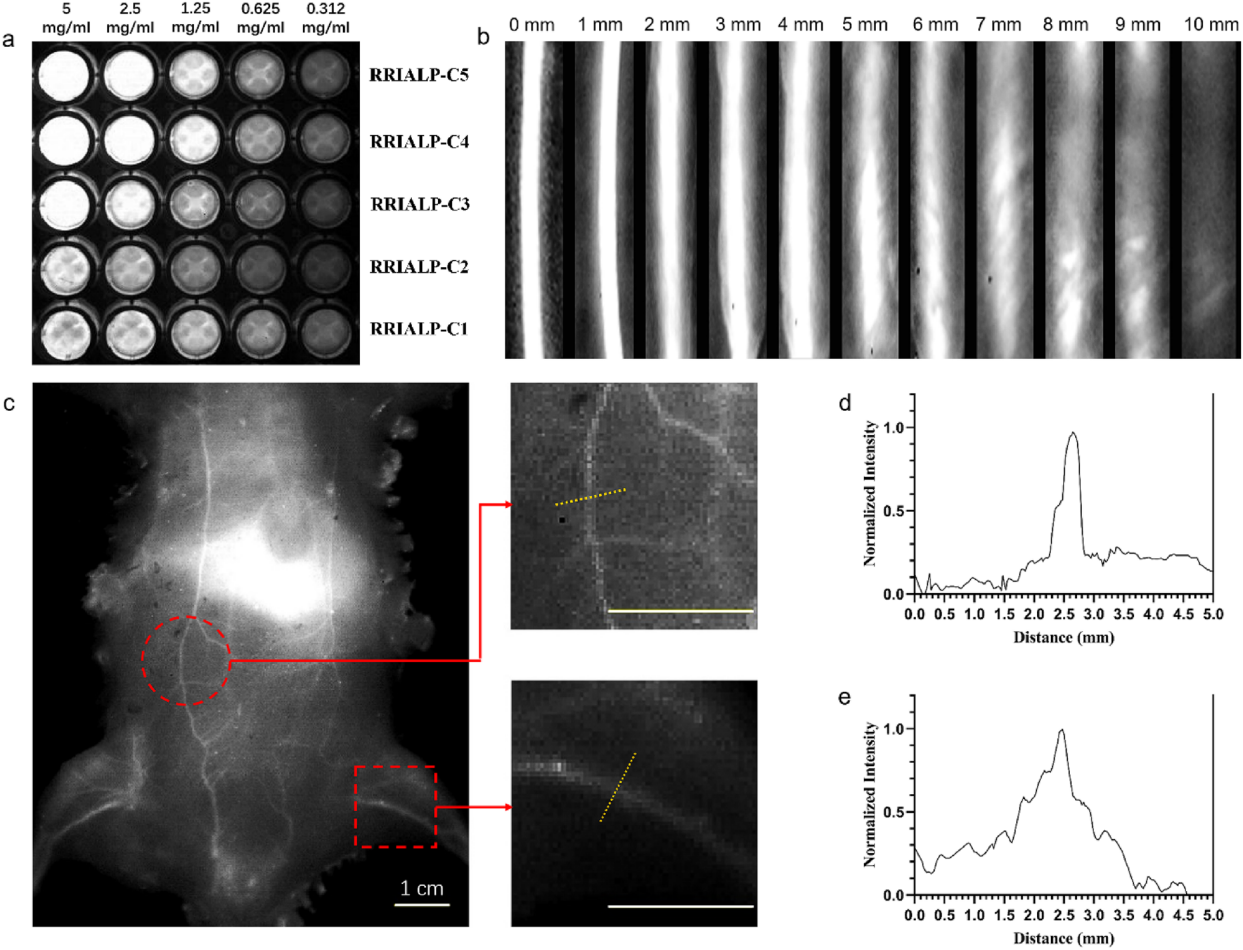
NIR-II FI properties of RRIALP-C4. **a** The NIR-II fluorescence signals of liposomes at different phospholipid concentrations. **b** The NIR-II fluorescence signals of liposomes are covered by different thicknesses of chicken tissues. **c** Systemic angiography of the Balb/c mice body after intravenous injection with RRIALP-C4 under 1064 nm laser excitation (1064 nm LP and 1064 nm OD filters). **d**, **e** Fluorescence intensity analysis of abdomen and hind limb vasculatures

Next, we investigated the NIR-II fluorescence signals of RRIALP-Cs in different phospholipid concentrations (Fig. 6a). Under the high concentrations (> 2.5 mg/mL), the fluorescence intensity of RRIALP-Cs tented to be no longer increased when the molar content of IR-1061 is over 1.5% because the maximum detection limit of the instrument was already reached. Moreover, under the low concentrations, the trend of fluorescence intensity enhancement would continue until the molar content of IR-1061 was 2% and then began to decrease, indicating that RRIALP-C4 possessed the best imaging effect for NIR-II FI. Next, the tissue penetration depth of RRIALP-C4 was also evaluated in vitro. As shown in Fig. 6b, RRIALP-C4 was able to maintain significant fluorescence intensity and spatial resolution within 6 mm, and even was detected over 9 mm, demonstrating a good tissue penetration depth.

The NIR-II vascular FI of the whole body was captured under the 1064 nm excitation after a tail vein injection of RRIALP-C4 (2 mg/mL) to evaluate its NIR-II FI in vivo performance. The systemic vascular structure of mice was discerned under a significant contrast with the surrounding background tissues (Fig. 6c). Furthermore, the fluorescence signal intensity of the abdominal and hind limb blood vessels of mice were analyzed (Fig. 6d, e). The full widths at half maximum (FWHM) of these vessels were calculated to be 0.4 and 0.6 mm, demonstrating that the NIR-II FI of RRIALP-C4 possessed extremely high spatial resolution in vivo.

### NIR-II FI and PTI of tumor

**Fig. 7:**
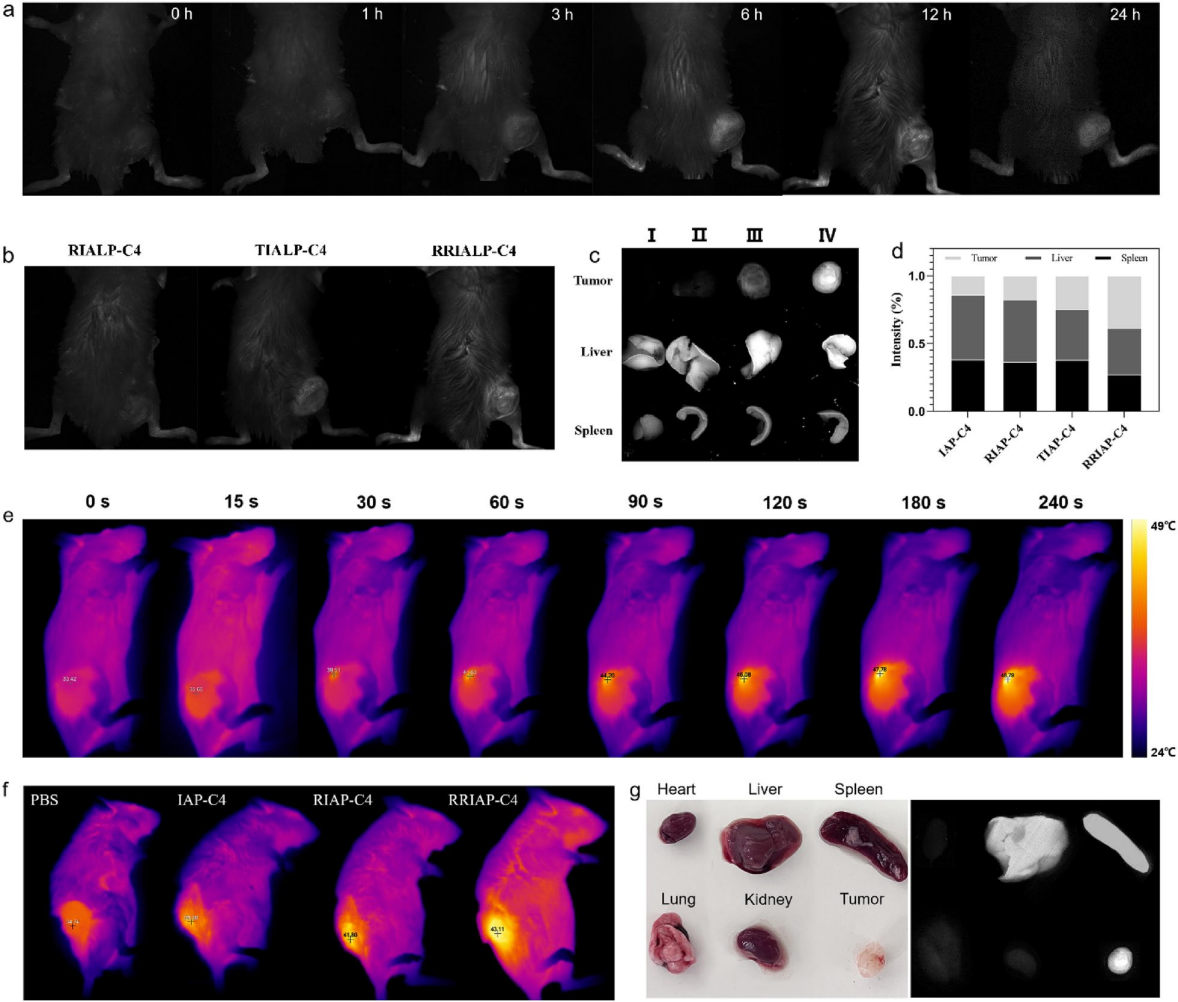
In vivo NIR-II FI and PTI of RRIALP-C4. **a** In vivo NIR-II FI of RRIALP-C4 at different time points (0, 1, 3, 6, 12, 24 h). **b** In vivo NIR-II FI of different liposomes after projection for 12 h. **c**, **d** Fluorescence intensities of different liposomes in liver, spleen, and tumor after projection for 12 h. I: IALP-C4. II: RIALP-C4. III: TIALP-C4. IV: RRIALP-C4. **e** In vivo PTI of RRIALP-C4 at different time points (0, 15, 30, 60, 90, 120, 180, 240 s). **f** In vivo PTI of different liposomes after irradiation for 240 s. **g** Distributions of RRIAP-C4 in different organs after treatment for 24 h

To investigate the influence of different peptides on the accumulation of IALP-C4 in tumor tissue, the prolonged fluorescent signal monitoring of mice was recorded. As shown in Fig. 7a, the fluorescence intensity of tumor tissue was lighted as soon as 3 h after injection of RRIALP-C4, then reached its maximum at 12 h and was able to maintain for 24 h. Compared with other IALP-C4 liposomes (Additional file 1: Fig. S17 and Fig. 7b), RRIALP-C4 showed the most rapid signal enhancement and the highest fluorescence intensity. Moreover, the fluorescence intensities of the liver, spleen, and *ex-vivo* tumor tissues of mice were captured (Fig. 7c, d). The results showed that IALP-C4 and RIALP-C4 were only slightly enriched in tumor tissue, which was much lower than in other tissues. The enrichment of TIALP-C4 in tumor tissue was the same as in the spleen, but still lower than in the liver. While RRIALP-C4 possessed the highest degree of enrichment in tumor tissue, almost the same as liver, indicating that RRIALP-C4 had a satisfactory tumor-targeting ability.

The temperature variation of tumor tissues was monitored using the thermal imager to examine the photothermal effect of liposomes on the tumor. Under the 808 nm laser (0.3 W/cm^2^) excitation, the tumor of mice treated with RRIALP-C4 was heated up rapidly and the temperature was up to 46 °C in 3 min. While the temperatures of tumor areas in other mice treated with RIALP-C4 or TIALP-C4 were hardly able to rise to an effective PTT temperature even though they were higher than that in the control group (Fig. 7e, f and Additional file 1: Fig. S15). These results demonstrated that RRIALP-C4 was an efficient liposome for both NIR-II FI and PTT of tumor tissue. In addition, the fluorescence images of normal organs (heart, liver, spleen, lung, and kidneys) and tumors of mice treated with RRIALP-C4 were harvested to evaluate its distribution in vivo (Fig. 7g). The results showed that RRIALP-C4 was mainly accumulated in the tumor and liver, followed by spleen, but not in heart, lung, and kidney.

### In vivo synergistic therapy of RRIALP-C4

**Fig. 8:**
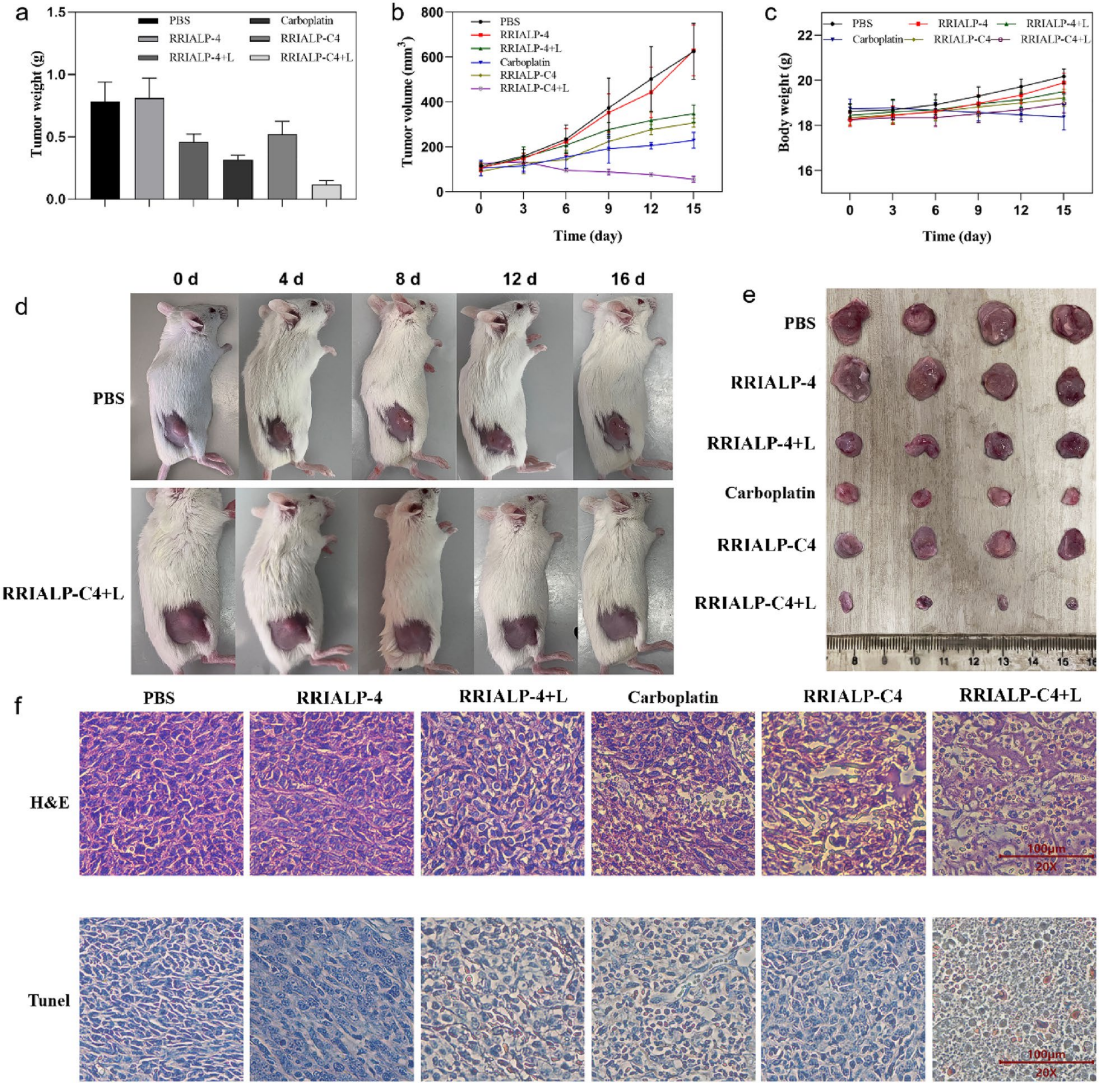
In vivo synergistic therapy of RRIALP-C4. **a** Tumor weight of different treatment groups after 15 days of treatment. **b** Tumor volume change curves of different treatment groups during the monitoring period. **c** Bodyweight change curves of mice in different treatment groups. **d** Images of tumor changes with the synergistic treatment of RRIAP-C4 for 15 days. **e** Tumor sizes of different treatment groups after 15 days of treatment. **f** H&E and Tunel staining of tumor sections after 15 days of treatment

Finally, the synergistic therapeutic efficacies of RRIALP-C4 were then carried out in the A549 cell transplanted tumor-bearing mice model. After 15 days of treatment, the changes in tumor volume and weight were recorded to evaluate the treatment effect (Fig. 8a, b). Mice treated with RRIALP-4 alone showed no treatment effect compared with that treated with PBS. The treatment of RRIALP-4 with irradiation only alleviated tumor growth to a certain extent, showing limited therapeutic effect. The treatments of the carboplatin group and RRIALP-C4 with irradiation group successfully inhibited the growth of the tumor, while the latter could even reduce tumor volume, indicating the synergistic therapy of RRIALP-C4 had the optimal tumor treatment effect. The monitoring of tumor changes in the treatment of RRIALP-4 with irradiation intuitively demonstrated the therapeutic efficacy compared with the treatment of PBS (Fig. 8d). Final tumor morphologies of different groups after 15 days of treatment were consistent with the previous results (Fig. 8e). By recording the weight of mice in different groups, there was negligible mice weight loss in other treatment groups except for the carboplatin alone group (Fig. 8c). Although the carboplatin treatment group can inhibit tumor growth, it also does some damage to the body of mice. Moreover, tissue sections of various organs after RRIALP-C4 injection showed no significant toxicity compared with the PBS group (Additional file 1: Fig. S16). The results demonstrated that RRIALP-C4 possessed excellent biosafety and negligible side effects in the whole treatment process. The histological morphology of the tumor tissues in different groups were shown in Fig. 8f. The group of RRIALP-C4 with irradiation showed a significant cell shrinkage and separation, which demonstrated the most severe tumor cell damage compared with other groups. The single drug-loaded liposome combi group (RRALP-C+RRIALP-4+L) and RR-ICG-ALP were also added as control group (Additional file 1: Fig. S17). The results showed that RR-ICG-ALP could alleviated tumor growth at beginning, but cannot be sustained. This because ICG possess poor photothermal stability and cannot repeat photothermal cycles. On the other hand, the RRALP-C+RRIALP-4+L also showed an inferior therapeutic efficacy than RRIALP-C4. This indicated that this group eventually recruited less IR-1061 and carboplatin in the tumor tissue, illustrating the emergence of a competitive relationship between these two liposomes. Moreover, to further reveal the mechanism of synergistic therapy, TUNEL staining of tumor tissues was performed to evaluate cell apoptosis [37]. Tumor cells appeared massively apoptotic in the treatment of RRIALP-4 with irradiation, whereas only slight apoptosis occurred in the treatment of RRIALP-C4 alone, indicating the different pathways of tumor therapy between PTT and carboplatin. The treatment of RRIALP-C4 with irradiation showed a similar apoptosis degree as the treatment of RRIALP-4 with irradiation, but with a more favorable tumor-suppressive effect, demonstrating that there is a synergistic effect between PTT and carboplatin.

## Conclusion

In summary, for the first time, we constructed a NIR-II fluorescence liposome (RRIALP-C4) loaded with small organic molecules to achieve both FI and PTT effects by the strategy of developing an ingenious structure of fluorophores that contains both the aggregated and free states. Comprehensive and in-depth research on the H-aggregated state of IR-1061 in ALPs was conducted, from theoretical mechanism to intracellular changes. We demonstrated that the narrow space of the phospholipid bilayer forced IR-1061 at high concentrations to accumulate face-to-face in liposomes with multilayer horizontal structure, thus resulting in the H-aggregated state. And this structure would not disappear after liposomes were phagocytized by cells. As ALPs were engulfed by A549 cells in the form of membrane fusion, IR-1061 was able to be transferred from liposomes to the cell membrane only, thus further maintaining its H-aggregated state. We believe this mechanism is universal, that is, using the characteristics of liposomes to modify the NIR-II fluorophore to make it have a variety of aggregated states is a new research direction, and has great application prospects.

The fluorescence and photothermal properties of RRIALP-C4 presented a dual-channel activated integration of intravital NIR-II FI under the excitation of 1061 nm and NIR-I PTT excited by 808 nm. To overcome the “stealth” characteristics of anionic liposomes, we enhanced the tumor-targeting ability of RRIALP-C4 through the surface charge modification and RGD targeting based on the investigation that charge modification would greatly enhance RRIALP-C4 entry into A549 cells. Moreover, RRIALP-C4 possessed the temperature-sensitive capability to ensure the release of encapsulated drug while producing the PTT effect, thus inducing synergistic treatment of thermochemotherapy. All these results demonstrated that RRIALP-C4 could achieve a great tumor accumulation and efficiently suppress tumor growth without any appreciable systemic toxicity. The rational design of RRIALP-C4 might open a new way in the development of organic small molecular NIR-II probes with the integration of precise tumor diagnosis by NIR-II FI and synergistic therapy dominated by PTT.

## Materials and methods

### Chemicals and materials

1,2-Dipalmitoyl-sn-glycero-3-phosphochol (DPPC), 1,2-dipalmitoyl-sn-glycero-3-phospho-(1-rac-glycerol) (DPPG), *N*-(carbonyl-methoxypolyethylene glycol 2000)-1,2-distearoyl-sn-glycerol-3-phosphoethanolamine (DSPE-mPEG_2000_) and cholesterol were obtained from Jiangsu Southeast Nanomaterials co., Ltd. (Jiangsu, China). Calcein-AM/PI Double Stain Kit, Annexin V-FITC/PI Apoptosis Detection Kit, Trypsin, and cell culture consumables were purchased from KeyGen Biological Technology Co., Ltd (Nanjing, China). Carboplatin, Rhodamine B (RhB), 3,3′-dioctadecyloxacarbocyanine perchlorate (DiO), IR-1061, and 3-(4,5-dimethyl-2-thiazolyl)-2,5-diphenyl tetrazolium bromide (MTT) were purchased from Sigma-Aldrich (Shanghai, China). HUVEC cells and A549 cells were obtained from the American Type Culture Collection (ATCC).

### Preparation of IR1061-ALP-Carboplatin (IALP-C)

Liposomes were prepared according to the injection method described in the previous literature [38]. In briefly, 10 mg DPPG, 10 mg DPPC, 3.38 mg cholesterol, 7.5 mg mPEG_2000_-DSPE and 0.2 mg IR-1061 were prepared and dissolved in a dichloromethane/ethanol mixture (2: 1, v/v). The mixed solution was added dropwise to deionized water dissolved with 1 mg carboplatin and stirred continuously under 50 °C for 30 min. After that, the residual organic solvent in the reaction solution was removed by rotary evaporation and the original liposome solution was supplemented to a phospholipid concentration of 10 mg/mL. Finally, the synthesized IALP-C was obtained after sonication (600 w, 20% amplitude) and filtration (PES syringe filter, 0.22 μm).

Similarly, the preparation process of IR1061-ALP (IALP) is identical to that of IALP-C except that carboplatin was not added. RhB-ALP (RALP) and DiO-ALP (DALP) were synthesized by replacing IR-1061 with equimolar RhB and DiO.

### Preparation of RR_9_-IR1061-ALP-Carboplatin (RRIALP-C)

1 mg RR_9_ was dissolved in 100 µL deionized water and then added to 1 mL IALP-C solution. After rotating at 4 °C for 4 h, the RRIALP-C nanoparticles were collected by ultrafiltration method for three times and re-dispersed in deionized water with a phospholipid concentration of 10 mg/mL for future experiments.

Similarly, the preparation process of RGD-IR1061-ALP-Carboplatin (RIALP-C) and TAT_48–60_-IR1061-ALP-Carboplatin (TIALP-C) is identical to that of RRIALP-C except for replacing RR_9_ with equimolar RGD and TAT_48–60_.

### The physicochemical characterization of liposomes

The morphology of liposomes was captured using the transmission electron microscope (JEM 1200EX, Japan) at 100 kV voltage. The mean hydrodynamic size and ζ-potential of liposomes were measured by a Malvern Zeta-sizer Nano instrument (Malvern Instruments Ltd, Malvern, UK).

The absorption spectra of liposomes were determined by a UV–Vis–NIR spectrophotometer (Shimadzu, Kyoto, Japan). The NIR-II fluorescence emission spectra were acquired using the low-temperature time-resolved fluorescence spectrometer FLS980 (Edinburgh Instruments, Edinburgh, UK) under the excitation of 980 nm.

The loading efficiency and release rate of drugs in liposomes were calculated by the content of residual drugs in the filtrate. The liposome solution after experiments was ultrafiltered and then the filtrate was taken to detect the absorbance. The concentration of IR-1061 and carboplatin was calculated by detecting the absorbance values of 1061 nm and 229 nm, respectively.

### Molecular dynamics simulations

Geometry optimization of IR-1061 was performed by Gaussian09 with the Becke three parameters hybrid exchange-correlation functional (B3LYP) and the standard Gaussian-type basis sets 6-311G(d). And the Restrained Electrostatic Potential (RESP) charge was calculated by Multiwfn. The D3 version of Grimme’s dispersion with Becke-Johnson (BJ) damping was added. The simulations were carried out with the GROMACS 2019.6 software package using the Amber force field. Isotropic pressure coupling was used to maintain a constant pressure of 1 atm, using Parrinello-Rahman barostat. The temperature was coupled to a constant of 298.15 K using the v-rescale thermostat. Periodic boundary conditions were applied in XY directions. The long-range electrostatic interactions were treated with the Particle-Mesh-Ewald (PME) method, and the van der Waals interactions were calculated with a cutoff distance of 12 Å. All bonds related to hydrogen were maintained constant at their equilibrium values with the Liner Constraint Solver (LINCS) algorithm and a time step of 1.0 fs was used. Water geometry was constrained using the SETTLE algorithm. Each system ran for 90 ns. The VMD software was used to analyze and visualize the simulation results.

### Evaluation of PTT of liposomes in vitro

The photothermal properties of liposome solution were measured by optical fiber thermometry. Specifically, liposomes with different concentrations were placed in 1 mL Eppendorf tubes and irradiated by an 808 nm laser (0.3 W/cm^2^) (Hi-Tech Optoelectronics Co., Ltd, China) for 10 min. The temperature of the solution was recorded in real-time by an optical fiber thermometer (UMI4, FISO, Canada) to make a temperature rise curve. Furthermore, a laser-off temperature change of the solution was also recorded to make a temperature drop curve.

The photothermal conversion efficiency (η) was calculated using Eqs. (1) and (2) expressed as follows.

1 $${\upeta}=\frac{\text{hS}({\text{T}}_{\text{max}}-{\text{T}}_{\text{suur}})-{\text{Q}}_{\text{dis}}}{\text{I}(1-{10}^{-{\text{A}}_{808}})}$$

2 $${{\uptau }}_{\text{s}}=\frac{{m}_{D}{C}_{D}}{hS}$$

The parameters S, h, T_max_, T_surr_, Q_dis_, I and A_808_ represent the container’s surface area, heat-transfer coefficient, maximum laser-trigger temperature, indoor temperature, heat dissipation caused by the light-absorbing of quartz cuvette, the intensity of laser (0.3 W/cm^2^) and absorbance of liposomes at 808 nm.

The parameters τ_s_, m_D,_ and C_D_ represent the time constant of the sample system, the mass, and the heat capacity of the solvent.

Moreover, the tissue penetration depth of the photothermal effect of liposomes was evaluated using chicken tissue in vitro. The chicken breast was cut into 1 mm thin slices and pasted on the sidewall of the Eppendorf tubes. The temperature rise curve of samples was then detected under the same conditions above.

In vitro, photothermal images of RRIALP-C4 were captured by the thermal imager (Ti32, Fluke, US). 1 mL RRIALP-C4 with different concentrations were placed into 24-well plates and irradiated from the top of the plate with 808 nm laser (0.3 W/cm^2^) and the temperatures were recorded.

### Evaluation of NIR-II FI properties of liposomes in vitro

NIR-II fluorescence intensity of RRIALP was detected by the small animal NIR-II biological imaging system (Wuhan grand Imaging Technology Co., Ltd., China). Specifically, liposomes with different concentrations were placed into 96-well plates and excited by a 1064 nm laser. The emitted fluorescence was captured by a NIR-II camera with a 1064 nm long-pass filter (1064 LP) and a 1064 nm cut-off filter (1064 OD) under an exposure time of 300 ms. The 1064 OD filter can cut off the wavelength at 1064 nm and avoid the background interference of the excitation wavelength.

The simulation experiment of tissue penetration depth in vitro was carried out using chicken tissue. Briefly, liposomes injected into a capillary tube were covered with the chicken breast which was cut into small pieces with uniform size but varying thickness. The fluorescence intensity of liposomes was then detected by the small animal NIR-II biological imaging system under the same condition above.

### Cytotoxicity assay

Cell viability was detected using MTT assays. A549 cells (1 × 10^4^ cells per well) were plated onto 96-well plates with 100 µL DMEM/F12 medium and cultured for 12 h in the CO_2_ incubator. Subsequently, 100 µL liposomes solution with different concentrations was added to the medium, and cells were incubated for a further 12 h. Afterward, the medium, in which 20 µL 3-(4,5-dimethylthiazol-2-yl)-2,5diphenyltetrazolium bromide (MTT, 5 mg/mL) as a pretreatment was added for 4 h, was then replaced with 150 µL DMSO and vibrated to dissolving crystallization. The absorbance of A549 cells was measured by a microplate reader (TECON infinite 200 Pro; Switzerland) at 490 nm.

The living/dead cell counts were valued by Calcein-AM/PI Double Stain Kit. Similar to the cell culture described above, A549 cells (1 × 10^5^ cells per well) were plated onto 6-well plates and 1 mL liposomes solution was added to the medium after 12 h of incubation. In addition, for photothermal treatment groups, A549 cells were irradiated from the top of the plate with an 808 nm laser (0.3 W/cm^2^) for additional 6 min after the addition of liposomes. The medium was then cultured for another 12 h and washed with PBS for three times. Cell staining was performed with the instructions of the kit. The staining of living/dead cells was observed by fluorescence inverted microscope (Olympus, Japan).

The A549 cell apoptosis was detected using Annexin V-FITC/PI Apoptosis Detection Kit and all experimental steps were almost consistent with the above [39]. After cell culture, A549 cells were collected using trypsin and washed with PBS three times. Cell staining was performed by the instructions of the kit and detected by flow cytometry (Thermo Scientific, USA).

The combinational index through the formula: $\text{CI}=\frac{{D}_{A}}{{ICX}_{A}}+\frac{{D}_{B}}{{ICX}_{B}}$. CI means combinational index. D_A_ and D_B_ means the concentration of combinational drug when cytostatic rate is X. ICX_A_ and ICX_B_ means the concentration of single drug when cytostatic rate is X.

### In vitro cellular uptake

The cellular uptake of liposomes was visualized by fluorescence staining. A549 cells (1.0 × 10^5^ cells per dish) were plated onto confocal dishes with 2 mL DMEM medium and cultured for 12 h in the CO_2_ incubator. After that, A549 cells were washed with PBS, and then 500 µL 1 mg/mL Hoechst solution, 200 µL 10 µmol/L DiO solution, and 200 µL 16 µg/mL RALPs were added separately and incubated for 5, 10, and 15 min, respectively. Cell staining was observed by laser confocal microscope (Nikon eclipse Ti2, Japan).

For quantitative analysis, A549 cells (1.0 × 10^5^ cells per well) were plated onto 6-well plates and collected using trypsin after being cultured for 12 h in the CO_2_ incubator. The collected cells were stained according to the above staining steps, and the fluorescence intensity was quantitatively detected by flow cytometry.

The membrane entry mechanism of liposomes was studied by observing the cell distribution of hydrophobic dyes. A549 cells (1.0 × 10^5^ cells per dish) were plated onto confocal dishes and stained by 500 µL 1 mg/mL Hoechst solution and 200 µL 16 µg/mL DALPs according to the above staining conditions after incubation for 12 h in a CO_2_ incubator. Cell staining was observed by a laser confocal microscope.

### In vivo NIR-II FI and PTI

All in vivo procedures were performed under a protocol approved by the Institutional Animal Care and Use Committee of Southeast University. All animal experiments have obtained the ethical approval of Southeast University (20,211,202,006). Studies were conducted in Balb/c female mice from Qinglongshan Biosciences (China). Tumor-bearing mice were developed by subcutaneous transplantation of A549 cells (1 × 10^7^ cells/mice) on mice (20 ± 2 g, 5–6 weeks).

In vivo NIR-II FI was performed by the small animal NIR-II biological imaging system. Specifically, when the tumor volume of mice was larger than 200 mm^3^, mice were anesthetized by isoflurane and depilated on the abdomen and tumor sites. Then 0.2 mL IALP-C4s (2.5 mg/mL) were injected intravenously before imaging. The NIR-II FI of mice was excited by a 1061 nm laser (40 mW/cm^2^) and captured by a NIR-II camera with 1064 LP and 1064 OD filters under an exposure time of 3000 ms. Vascular imaging of mice was imaged immediately after liposome injection, and tumors were imaged for prolonged monitoring.

In vivo PTI was captured using the thermal imager. Mice were anesthetized by intraperitoneal injection of 7% chloral hydrate (5 mL/kg) and depilated on the tumor sites. Then 0.2 mL liposome solution (2.5 mg/mL) was injected intravenously before imaging. Mice were irradiated under 808 nm laser (0.3 W/cm^2^) and the temperature changes at tumor sites were recorded.

### Biodistribution assay

The biodistribution of liposomes can be evaluated by the NIR-II fluorescence intensity of each organ. After intravenous injection of liposomes for 24 h, mice were dissected and their heart, liver, spleen, lung, kidney, and tumor were removed. The liposome content of each organ was qualitatively analyzed by fluorescence intensity detection (excitation: 1064 nm, exposure time: 300 ms).

### In vivo synergistic therapy

When the tumor volume of mice was larger than 100 mm^3^, mice were randomly divided into six groups (n = 4 for each group): PBS, PBS+L, RRIALP-4, RRIALP-4+L, RRIALP-C4, RRIALP-C4+L (L represents 808 nm laser). The experimental groups were given the corresponding drugs and laser irradiation respectively. Specifically, after intravenous injection of 0.2 mL liposome solution (2.5 mg/mL) for 24 h, mice were exposed to the 808 nm laser on the tumor site for 5 min with a power density of 0.3 W/cm^2^. Mice were dosed and irradiated every 3 days for a total of 15 days, and the body weight and tumor size were recorded every time [40, 41]. After treatment, these mice were sacrificed, and their organs and tumors were collected. The collected tumors were fixed in 4% neutral-buffered paraformaldehyde and embedded in paraffin for hematoxylin–eosin (H&E) staining, and the images of the histological tumor sections were obtained. To investigate the damage degree of tumor tissues, terminal deoxynucleotidyl transferase dUTP nick end labeling (TUNEL) staining of tumors in each group was carried out after tissue sections and labeled with biotin.

### Data analysis

Data are usually set in three parallel groups and expressed as mean ± standard deviation (SD). In vitro NIR-II fluorescence intensities were measured by the small animal NIR-II biological imaging system, aligned, and analyzed by GraphPad Prism software. The data of flow cytometry were processed by Flowjo software. Analyses of in vitro and in vivo fluorescence images were performed using ImageJ software. The SBR was measured and quantified by grayscale analysis. Statistical analysis was carried out using analysis of variance followed by the unpaired-samples Student t test. Values of P < 0.05 were considered to indicate statistical significance.

## Supplementary Information

**Additional file 1: Figure S1.** Absorption and fluorescence spectra of IALP-C4,RIALP-C4, TIALP-C4 and RRIALP-C4. **FigureS2.** The H-aggregated state of IR-1061 in the system after running for 90 ns(dash line: distribution layers of IR-1061 in H-aggregated state). (a) Theratio of IR-1061 to DPPG is 1:10. (b) The ratio of IR-1061 to DPPG is 1:20. **Figure S3.** The linear fitting of − ln^θ^ and time in the cooling curve and photothermal conversionefficiency. (a) IALP-1. (b) IALP-2. (c) IALP-3. (d) IALP-5. (e) Photothermalstability of ICG-ALP (laser on/off for 4 consecutive cycles) irradiated with808 nm laser at 0.3 W/cm^2^. **FigureS4.** The photothermal conversion efficiency and photothermal stability ofIR-780-loaded liposomes (phospholipid concentration: 10 mg/mL). (a) The linearfitting of − ln^θ^ and time in the cooling curve andphotothermal conversion efficiency. (b) Photothermal stability of IR-780-loadedliposomes (laser on/off for 4 consecutive cycles) irradiated with 808 nm laserat 0.3 W/cm^2^. **Figure S5.** Theabsorption of RRIALP-C4 (phospholipid concentration: 10 mg/mL) after beingirradiated with 808 nm laser (0.3 W/cm^2^) for different cycles. **Figure S6.** (a) Thermal images of RRIALP-C4with different phospholipid concentrations after being irradiated with 808 nmlaser at 0.3 W/cm^2^ in 24-well plates (diluted with cell culturemedium). (b) Temperature changes of RRIALP-C4 with different phospholipid concentrations.**Figure S7.**Cell viability after drug treatment. (a) ICG aqueoussolution with different concentrations. (b) ICG-loadedliposomes (2% molar content) with different phospholipidconcentrations. **Figure S8.** In vitro photothermaltherapy and synergistic treatment for A549 cells. (a) Cytotoxicity of A549 cells treated with drugs group (6.7 μM Carbo+1mg/mL IALP-4+L) and single liposomes combi group (1mg/mL ALP-C+1mg/mL IALP+L) aftertreatment for 24 h. (b) Flow cytometry of apoptosis cells treated withdifferent conditions. Q1, Q2, Q3, and Q4 represent necrotic, lateapoptotic, early apoptotic, and viable cells, respectively. (c) Fluorescent staining of dead/living cellstreated with different conditions. Green channel: viable cells. Red channel:dead cells. **Figure S9.** (a) CLSMimages (100× oil) of A549 cells treated with 2 mg/mL RALP, RRALP, and TRALP for30 min. Blue channel: nucleus. Green channel: cell membrane. Red channel:liposomes. (b) CLSM images (100× oil) of A549 cells treated with 2 mg/mL DALP,RDALP, and TDALP for 30 min. Blue channel: nucleus. Green channel: liposomes.(c) CLSM images (100× oil) of A549 cells treated with 2 mg/mL DRALP, RDRALP,and TDRALP for 30 min. Blue channel: nucleus. Green and red channels:liposomes. **Figure S10. **Fluorescenceproperties of different phospholipid concentrationsof DiO-ALP and Rhb-ALP. (a) and (b) Excitation and emission spectra of DiO-ALP. (c) Maximum emission peak of DiO-ALP.(d) and (e) Excitation and emission spectra of Rhb-ALP. (f) Maximum emissionpeak of Rhb-ALP. **Figure S11.** Thermalimages of A549 cells treated with RRIALP-4 (2 mg/mL) in different times (irradiatedwith 808 nm laser at 0.3 W/cm^2^ for 5 min). **Figure S12.** Thermal images of A549 cells treated with ICG aqueoussolution and ICG-ALP in different times. (a) Top: ICG aqueous solution (50 μg/mL);Bottom: 2% molar content ICG-ALP (phospholipid concentration: 2 mg/mL). (b) and(c) Temperature change curves of ICG aqueous solution and ICG-ALP. **Figure S13.** Fluorescence images of A549cells phagocytosis of liposomes. (a) A549 cells treated with DRALP, RRDRALP,and RRDRALP+Pitstop 2 for 30 min. (b) Fluorescence analysis of RhB in A549 cells. **Figure S14.** (a) In vivo NIR-II FI of RIALP-C4 at different timepoints (0, 1, 3, 6, 12, 24 h). (b) In vivo NIR-II FI of TIALP-C4 at differenttime points (0, 1, 3, 6, 12, 24 h). **FigureS15.** (a) In vivo PTI of IALP-C4 at different time points (0, 15, 30, 60,90, 120, 180, 240 s). (b) In vivo PTI of RIALP-C4 at different time points (0,15, 30, 60, 90, 120, 180, 240 s). (c) In vivo PTI of TIALP-C4 at different timepoints (0, 15, 30, 60, 90, 120, 180, 240 s). **Figure S16.** H&E staining sections of organs from differentgroups. **Figure S17.** In vivo synergistic therapy of RR-ICG-ALP andsingle liosome combi group (RRALP-C+RRIALP-4+L). **Figure S18.** Absorption peaks of purified cell membrane after Incubated with IALP-4.

## Data Availability

The datasets used and/or analyzed during the current study are available from the corresponding author on reasonable request.
