# Supplementary material for: Transmissible H-aggregated NIR-II fluorophore to the tumor cell membrane for enhanced PTT and synergistic therapy of cancer
Source: Nano Converg. 2023 Jan 6;10:3. doi: 10.1186/s40580-022-00352-4 (PMC9823176; doi:10.1186/s40580-022-00352-4)
Supplement: Supplementary file 1 — Additional file 1: Figure S1. Absorption and fluorescence spectra of IALP-C4,RIALP-C4, TIALP-C4 and RRIALP-C4. FigureS2. The H-aggregated state of IR-1061 in the system after running for 90 ns(dash line: distribution layers of IR-1061 in H-aggregated state). (a) Theratio of IR-1061 to DPPG is 1:10. (b) The ratio of IR-1061 to DPPG is 1:20. Figure S3. The linear fitting of − lnθ and time in the cooling curve and photothermal conversionefficiency. (a) IALP-1. (b) IALP-2. (c) IALP-3. (d) IALP-5. (e) Photothermalstability of ICG-ALP (laser on/off for 4 consecutive cycles) irradiated with808 nm laser at 0.3 W/cm2. FigureS4. The photothermal conversion efficiency and photothermal stability ofIR-780-loaded liposomes (phospholipid concentration: 10 mg/mL). (a) The linearfitting of − lnθ and time in the cooling curve andphotothermal conversion efficiency. (b) Photothermal stability of IR-780-loadedliposomes (laser on/off for 4 consecutive cycles) irradiated with 808 nm laserat 0.3 W/cm2. Figure S5. Theabsorption of RRIALP-C4 (phospholipid concentration: 10 mg/mL) after beingirradiated with 808 nm laser (0.3 W/cm2) for different cycles. Figure S6. (a) Thermal images of RRIALP-C4with different phospholipid concentrations after being irradiated with 808 nmlaser at 0.3 W/cm2 in 24-well plates (diluted with cell culturemedium). (b) Temperature changes of RRIALP-C4 with different phospholipid concentrations.Figure S7.Cell viability after drug treatment. (a) ICG aqueoussolution with different concentrations. (b) ICG-loadedliposomes (2% molar content) with different phospholipidconcentrations. Figure S8. In vitro photothermaltherapy and synergistic treatment for A549 cells. (a) Cytotoxicity of A549 cells treated with drugs group (6.7 μM Carbo+1mg/mL IALP-4+L) and single liposomes combi group (1mg/mL ALP-C+1mg/mL IALP+L) aftertreatment for 24 h. (b) Flow cytometry of apoptosis cells treated withdifferent conditions. Q1, Q2, Q3, and Q4 represent necrotic, lateapoptotic, early apoptoti [file 40580_2022_352_MOESM1_ESM.docx]

**Additional file 1**

**Transmissible H-aggregated NIR-II fluorophore to the tumor cell membrane for enhanced PTT and synergistic therapy of cancer**

Haoli Yu^1^, Yuesong Wang^1^, Yan Chen^1^, Mengyuan Cui^1^, Fang Yang^1, *^, Peng Wang^2, *^, Min Ji^1, *^

^1^State Key Laboratory of Bioelectronics, Jiangsu Laboratory for Biomaterials and Devices, School of Biological Science and Medical Engineering, Southeast University, Nanjing, 210096, China

^2^Department of Biomedical Engineering, School of Engineering, China Pharmaceutical University, Nanjing, 210009, China.

Author for correspondence:

Min Ji, 101010516@seu.edu.cn

Peng Wang, [wangpeng159seu@hotmail.com](mailto:wangpeng159seu@hotmail.com)

Fang Yang, yangfang2080@seu.edu.cn


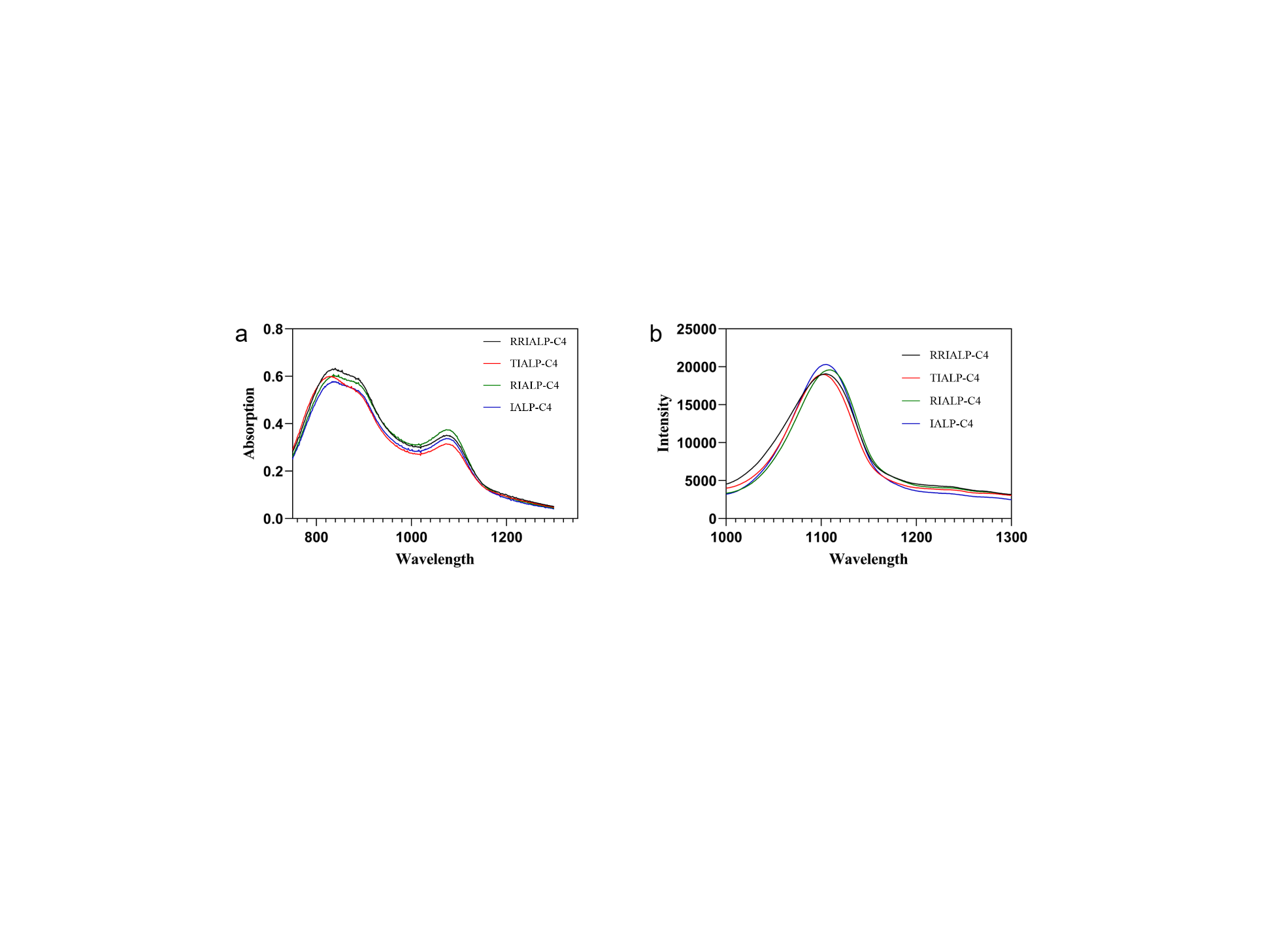


**Fig. S1.** Absorption and fluorescence spectra of IALP-C4, RIALP-C4, TIALP-C4 and RRIALP-C4.


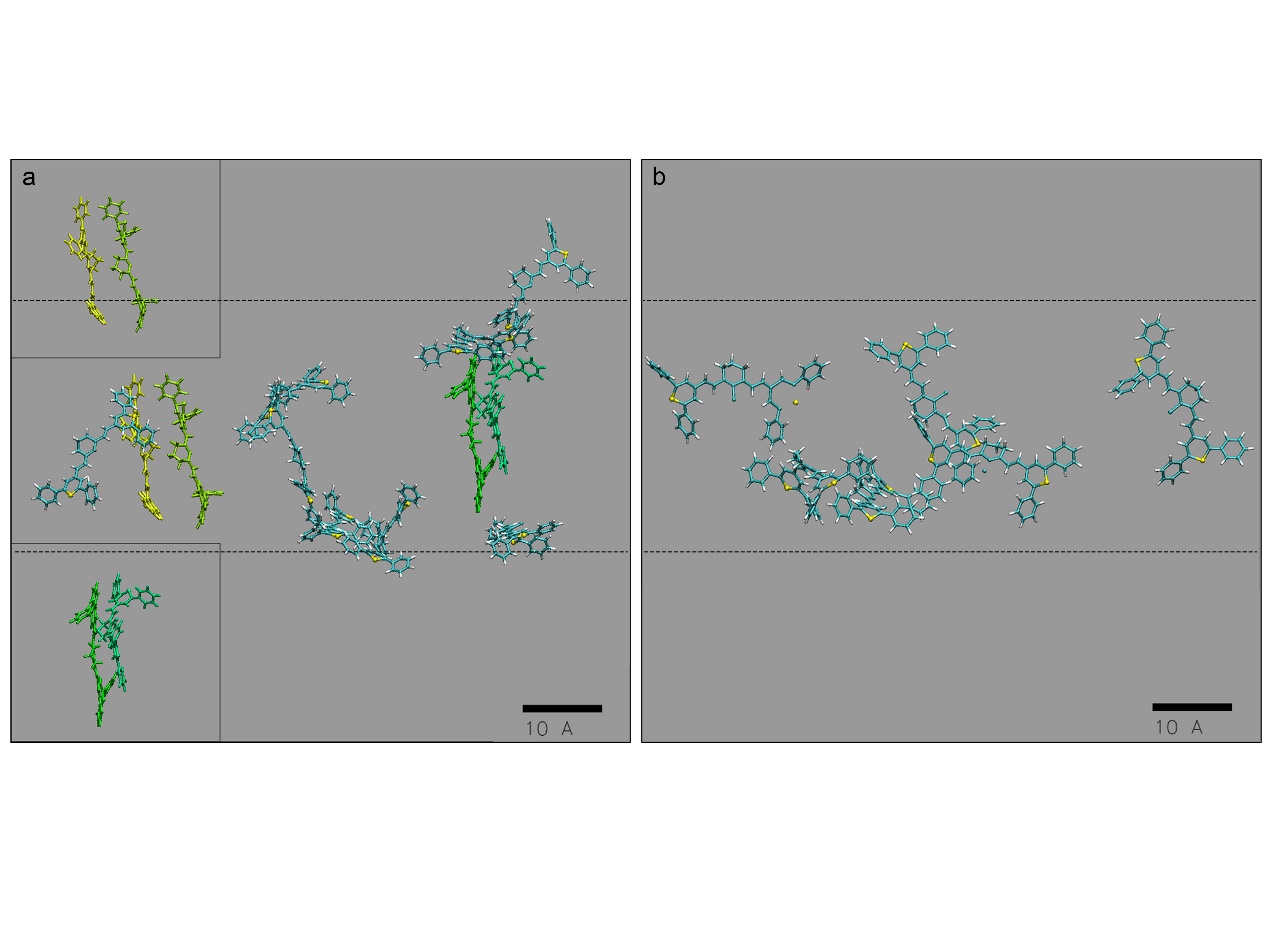


**Fig. S2.** The H-aggregated state of IR-1061 in the system after running for 90 ns (dash line: distribution layers of IR-1061 in H-aggregated state). (a) The ratio of IR-1061 to DPPG is 1:10. (b) The ratio of IR-1061 to DPPG is 1:20.


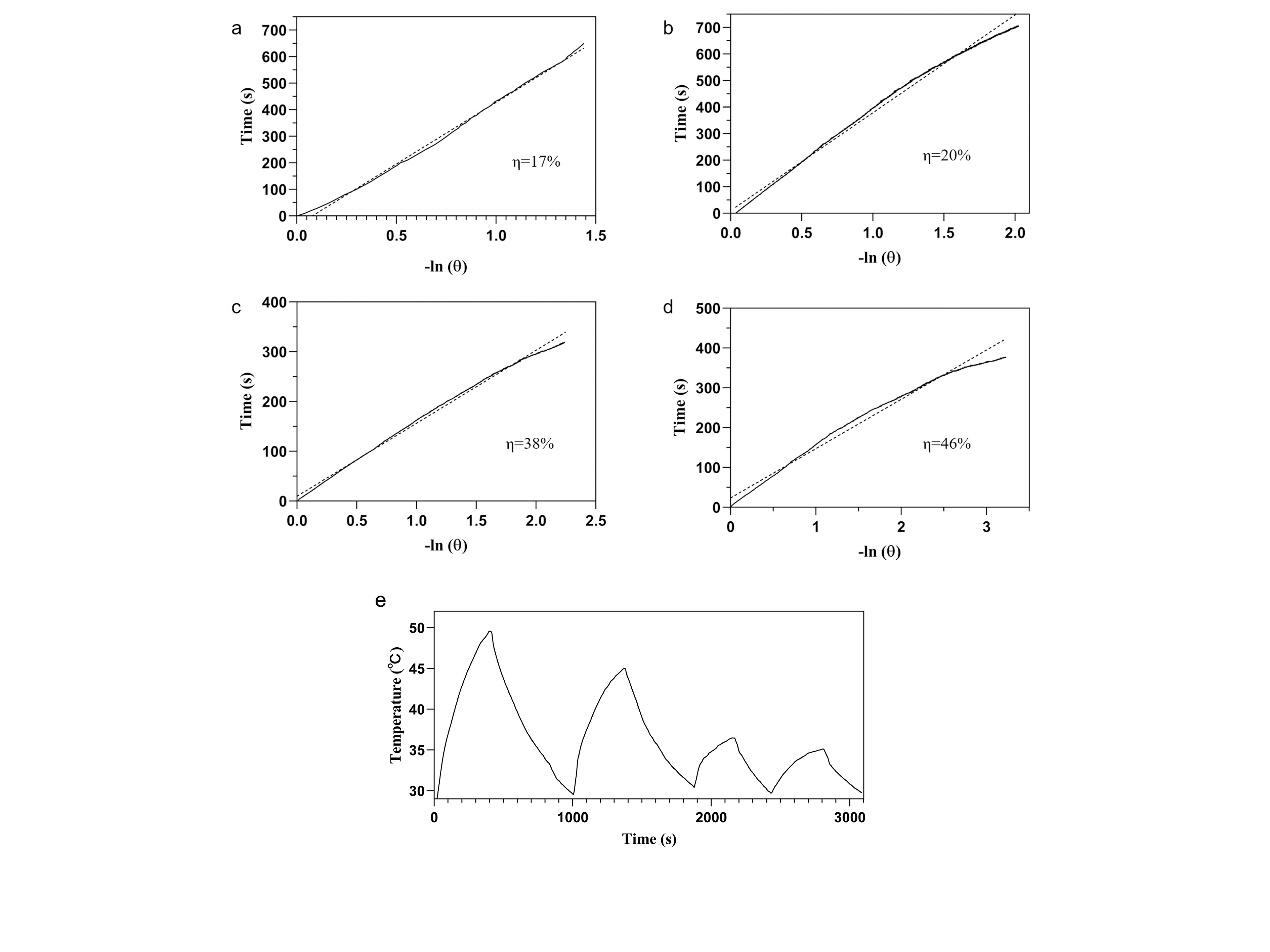


**Fig. S3.** The linear fitting of −ln^θ^ and time in the cooling curve and photothermal conversion efficiency. (a) IALP-1. (b) IALP-2. (c) IALP-3. (d) IALP-5. (e) Photothermal stability of ICG-ALP (laser on/off for 4 consecutive cycles) irradiated with 808 nm laser at 0.3 W/cm^2^.


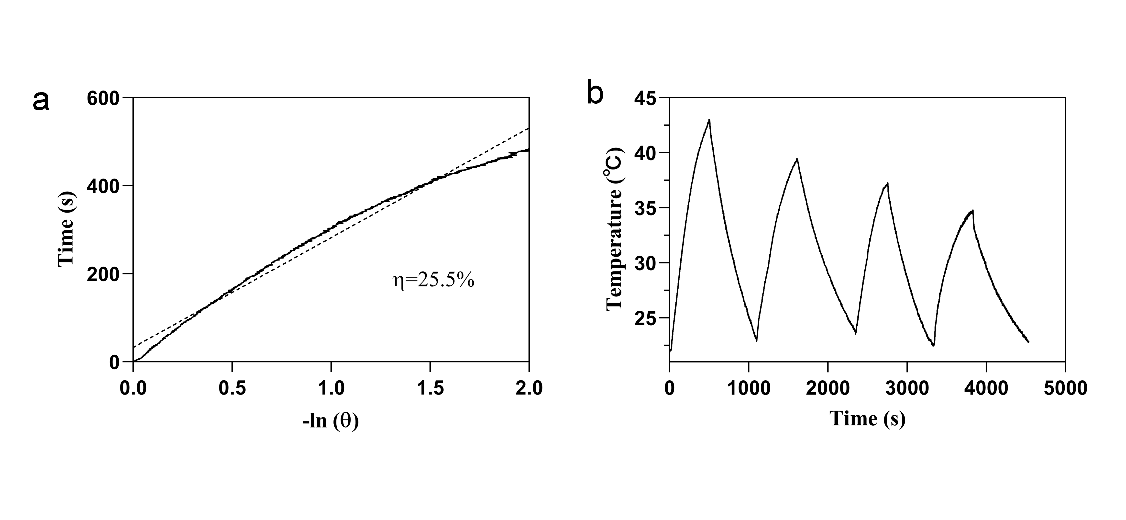


**Fig. S4.** The photothermal conversion efficiency and photothermal stability of IR-780-loaded liposomes (phospholipid concentration: 10 mg/mL). (a) The linear fitting of −ln^θ^ and time in the cooling curve and photothermal conversion efficiency. (b) Photothermal stability of IR-780-loaded liposomes (laser on/off for 4 consecutive cycles) irradiated with 808 nm laser at 0.3 W/cm^2^.


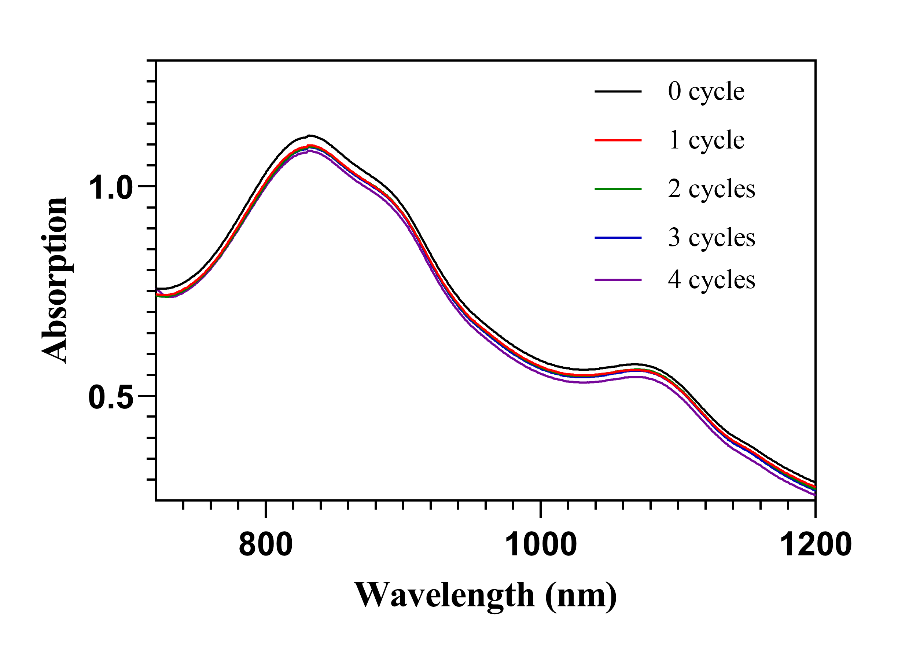


**Fig. S5.** The absorption of RRIALP-C4 (phospholipid concentration: 10 mg/mL) after being irradiated with 808 nm laser (0.3 W/cm^2^) for different cycles


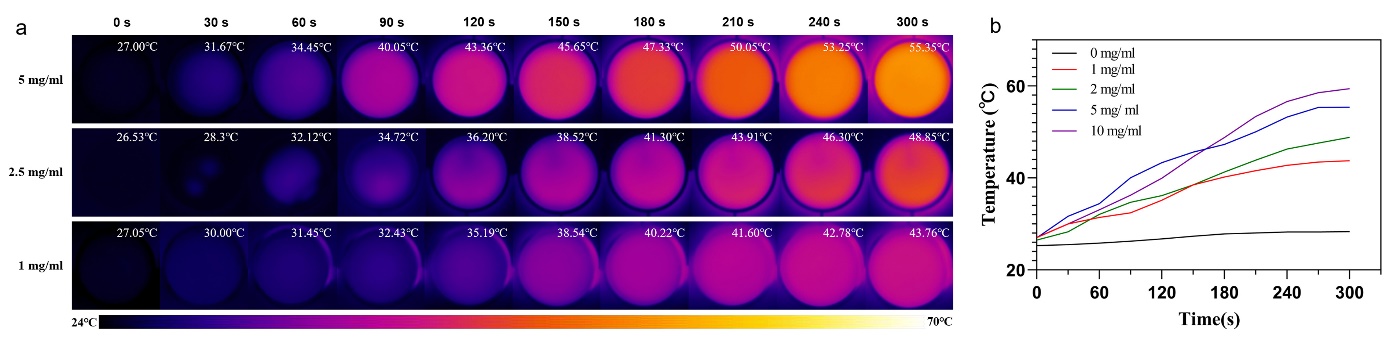


**Fig. S6.** (a) Thermal images of RRIALP-C4 with different phospholipid concentrations after being irradiated with 808 nm laser at 0.3 W/cm^2^ in 24-well plates (diluted withcell culture medium). (b) Temperature changes of RRIALP-C4 with different phospholipid concentrations.


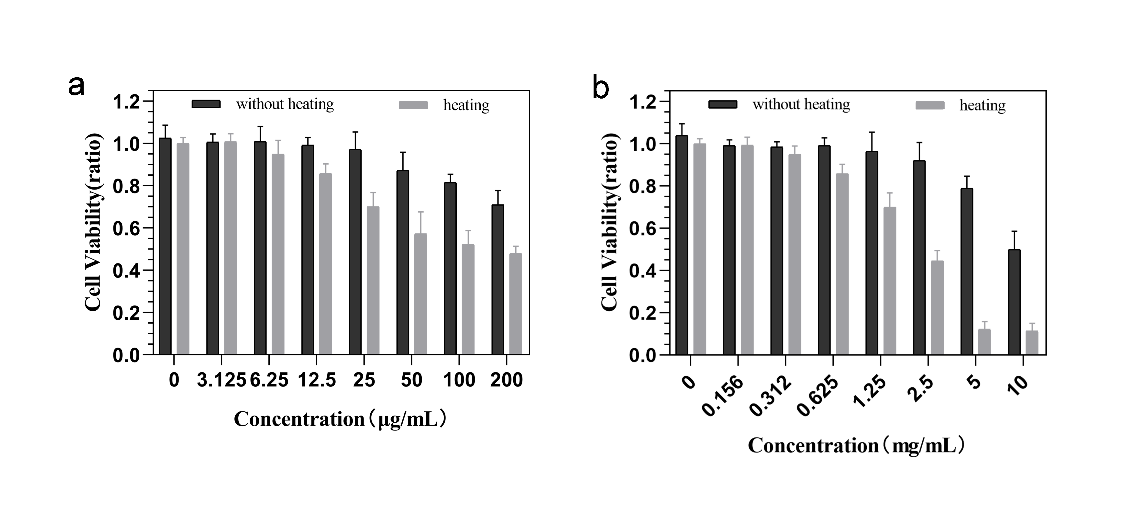


**Fig. S7.** Cell viability after drug treatment. (a) ICG aqueous solution with different concentrations. (b) ICG-loaded liposomes (2% molar content) with different phospholipid concentrations.


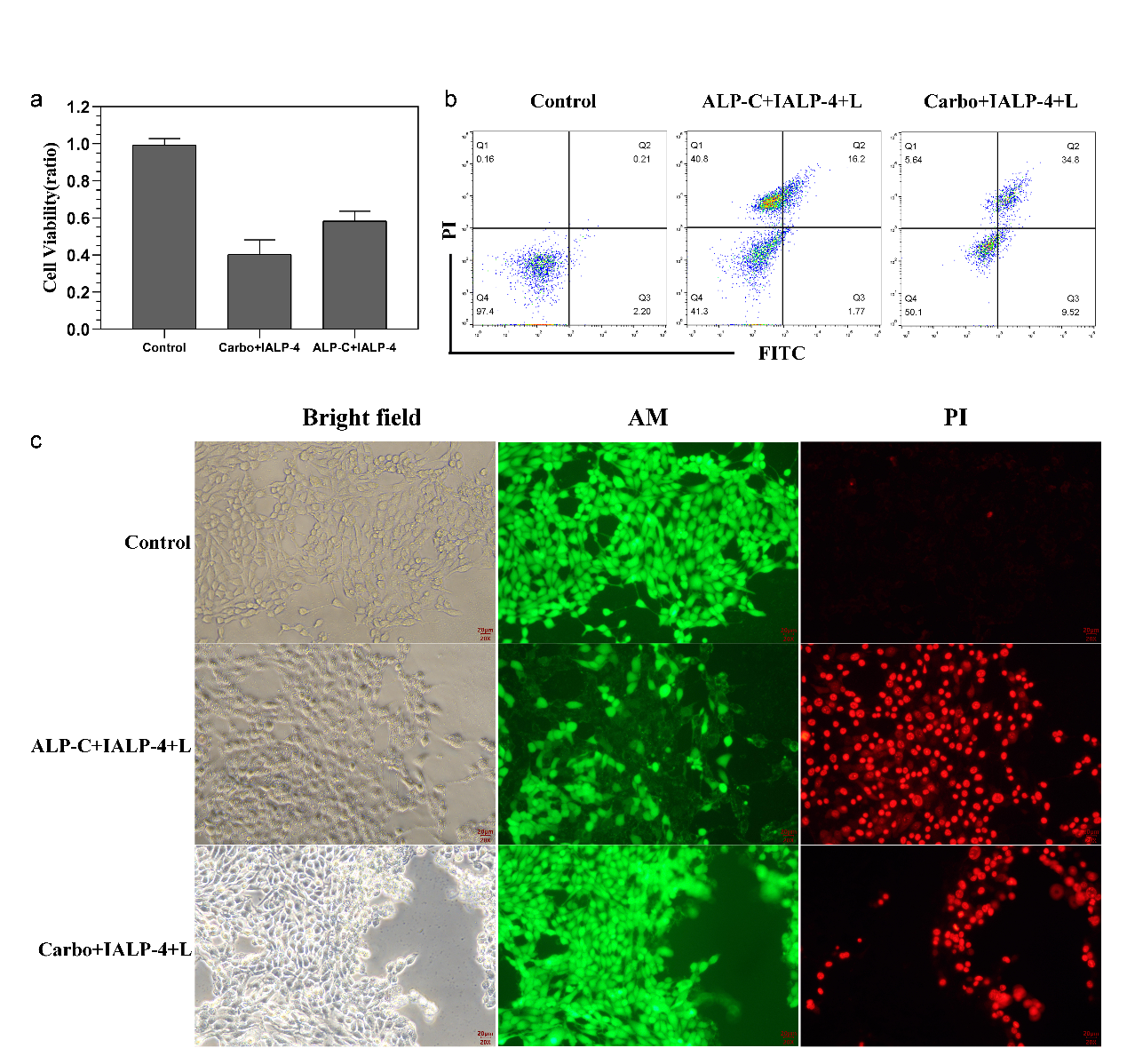


**Fig. S8.** *In vitro* photothermal therapy and synergistic treatment for A549 cells. (a) Cytotoxicity of A549 cells treated with drugs group (6.7 μM Carbo+1 mg/mL IALP-4+L) and single liposomes combi group (1mg/mL ALP-C+1mg/mL IALP+L) after treatment for 24 h. (b) Flow cytometry of apoptosis cells treated with different conditions. Q1, Q2, Q3, and Q4 represent necrotic, late apoptotic, early apoptotic, and viable cells, respectively. (c) Fluorescent staining of dead/living cells treated with different conditions. Green channel: viable cells. Red channel: dead cells.


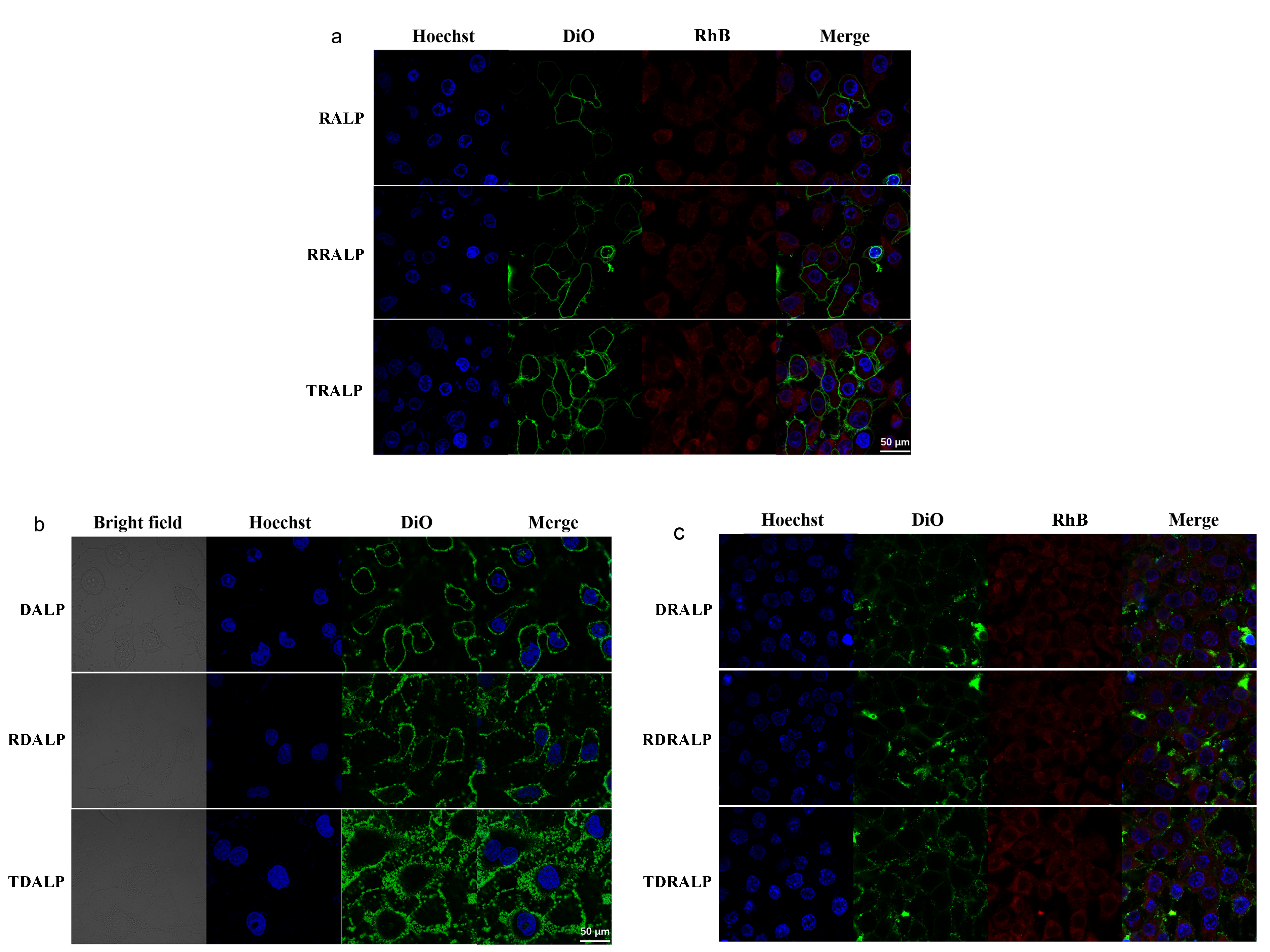


**Fig. S9.** (a) CLSM images (100× oil) of A549 cells treated with 2 mg/mL RALP, RRALP, and TRALP for 30 min. Blue channel: nucleus. Green channel: cell membrane. Red channel: liposomes. (b) CLSM images (100× oil) of A549 cells treated with 2 mg/mL DALP, RDALP, and TDALP for 30 min. Blue channel: nucleus. Green channel: liposomes. (c) CLSM images (100× oil) of A549 cells treated with 2 mg/mL DRALP, RDRALP, and TDRALP for 30 min. Blue channel: nucleus. Green and red channels: liposomes.


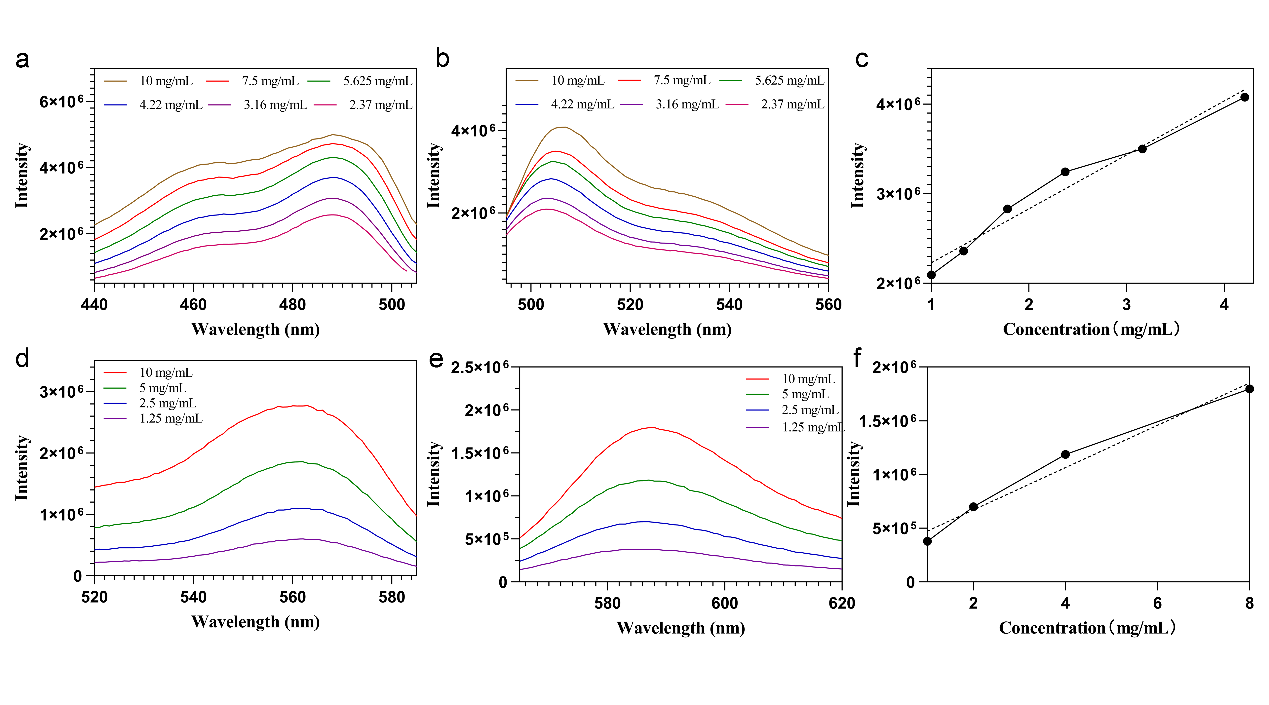


**Fig. S10.** Fluorescence properties of different phospholipid concentrations of DiO-ALP and Rhb-ALP. (a) and (b) Excitation and emission spectra of DiO-ALP. (c) Maximum emission peak of DiO-ALP. (d) and (e) Excitation and emission spectra of Rhb-ALP. (f) Maximum emission peak of Rhb-ALP.


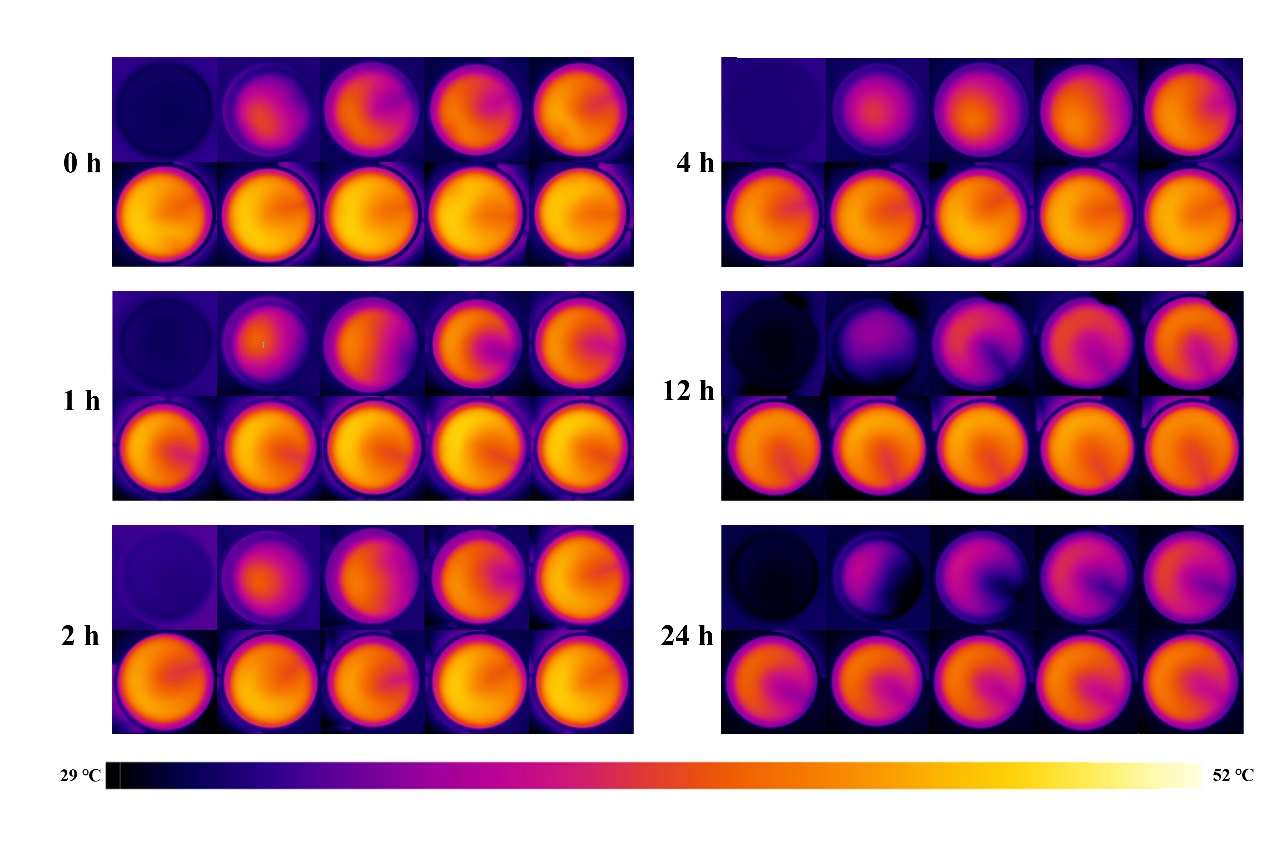


**Fig. S11.** Thermal images of A549 cells treated with RRIALP-4 (2 mg/mL) in different times (irradiated with 808 nm laser at 0.3 W/cm^2^ for 5 min).


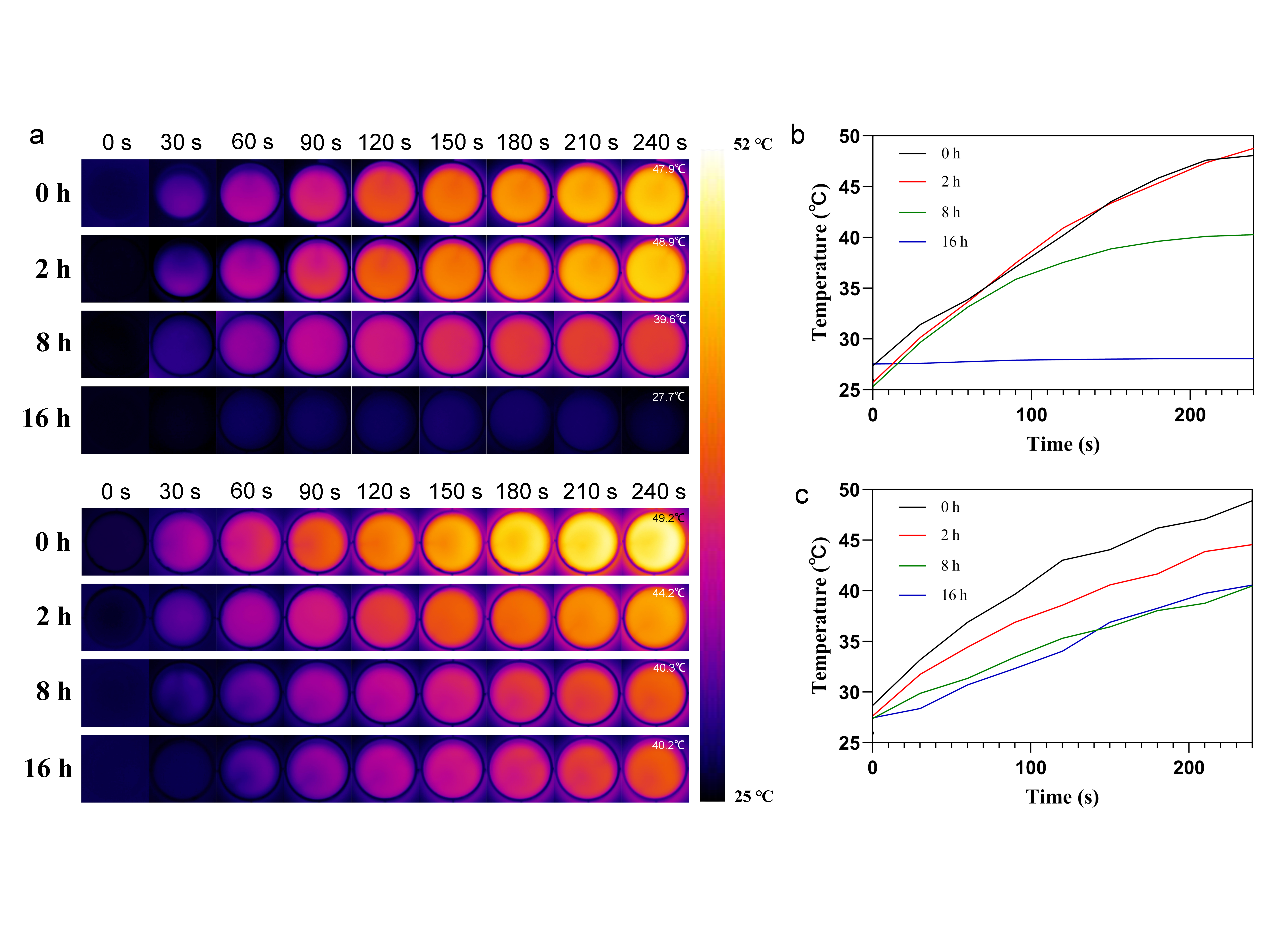


**Fig. S12.** Thermal images of A549 cells treated with ICG aqueous solution and ICG-ALP in different times. (a) Top: ICG aqueous solution (50 μg/mL); Bottom: 2% molar content ICG-ALP (phospholipid concentration: 2 mg/mL). (b) and (c) Temperature change curves of ICG aqueous solution and ICG-ALP.


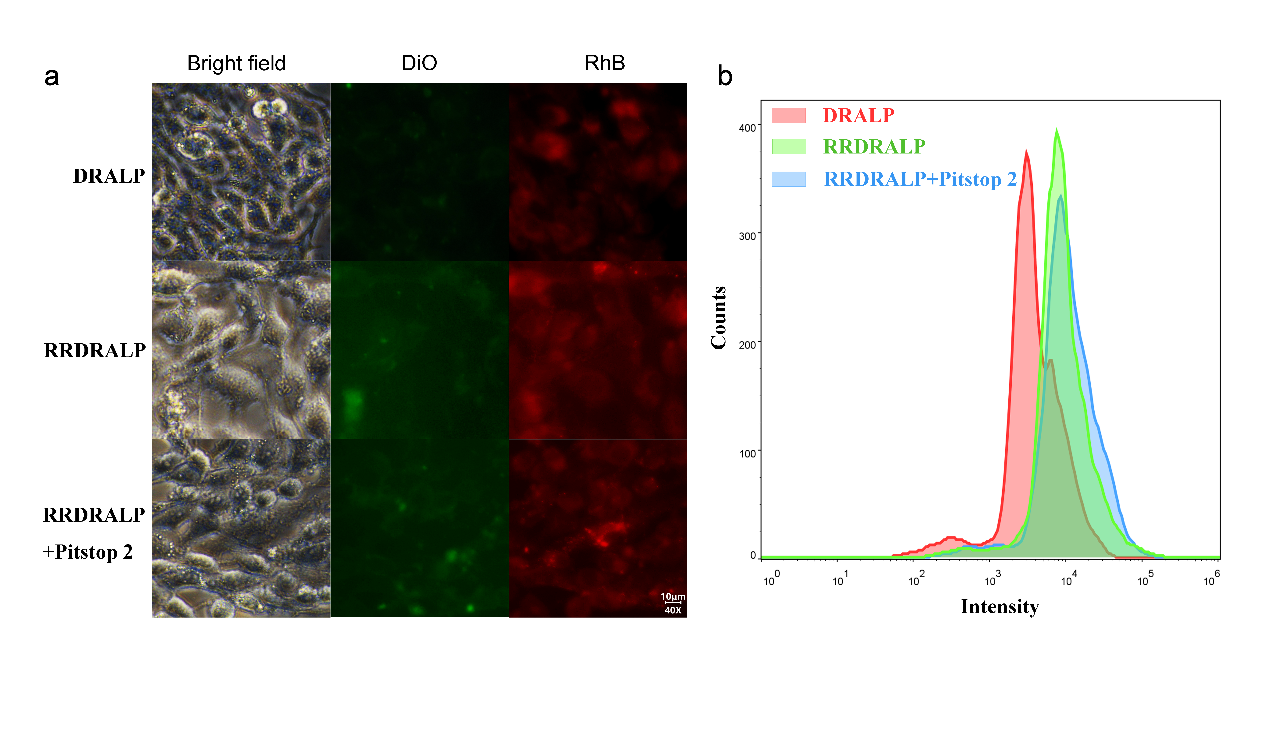


**Fig. S13.** Fluorescence images of A549 cells phagocytosis of liposomes. (a) A549 cells treated with DRALP, RRDRALP, and RRDRALP+Pitstop 2 for 30 min. (b) Fluorescence analysis of RhB in A549 cells.


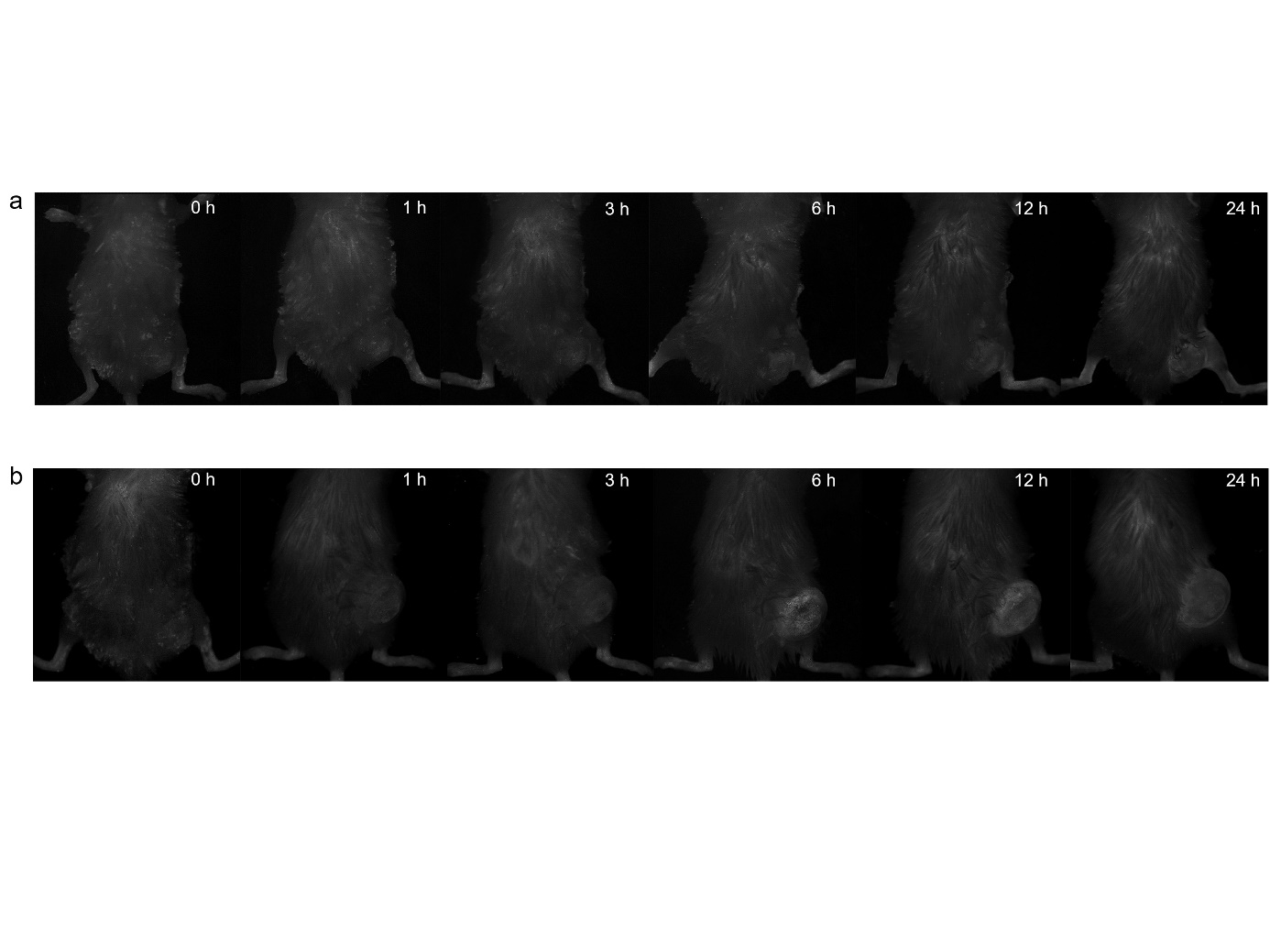


**Fig. S14.** (a) In vivo NIR-II FI of RIALP-C4 at different time points (0, 1, 3, 6, 12, 24 h). (b) In vivo NIR-II FI of TIALP-C4 at different time points (0, 1, 3, 6, 12, 24 h).


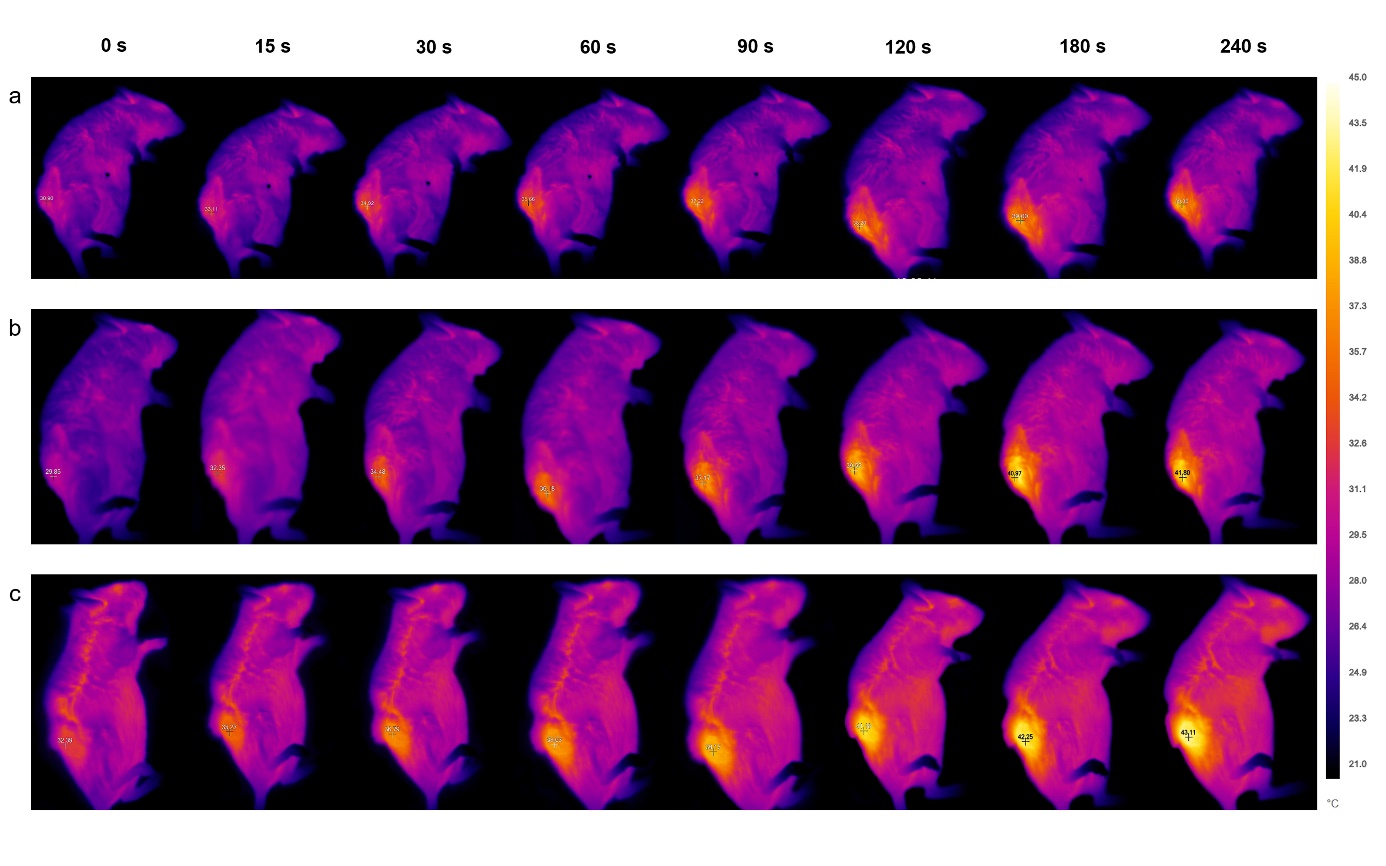


**Fig. S15.** (a) In vivo PTI of IALP-C4 at different time points (0, 15, 30, 60, 90, 120, 180, 240 s). (b) In vivo PTI of RIALP-C4 at different time points (0, 15, 30, 60, 90, 120, 180, 240 s). (c) In vivo PTI of TIALP-C4 at different time points (0, 15, 30, 60, 90, 120, 180, 240 s).


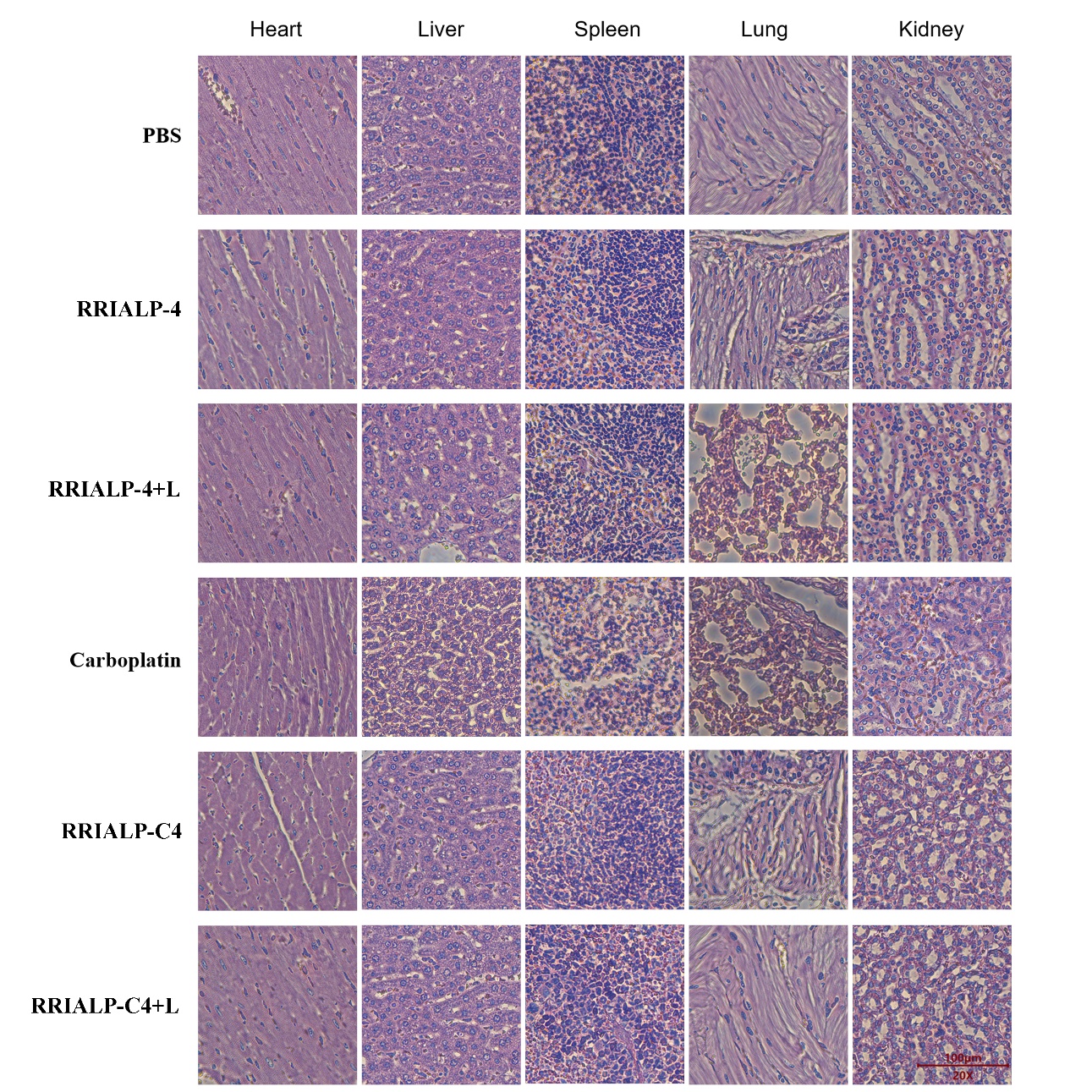


**Fig. S16.** H&E staining sections of organs from different groups.


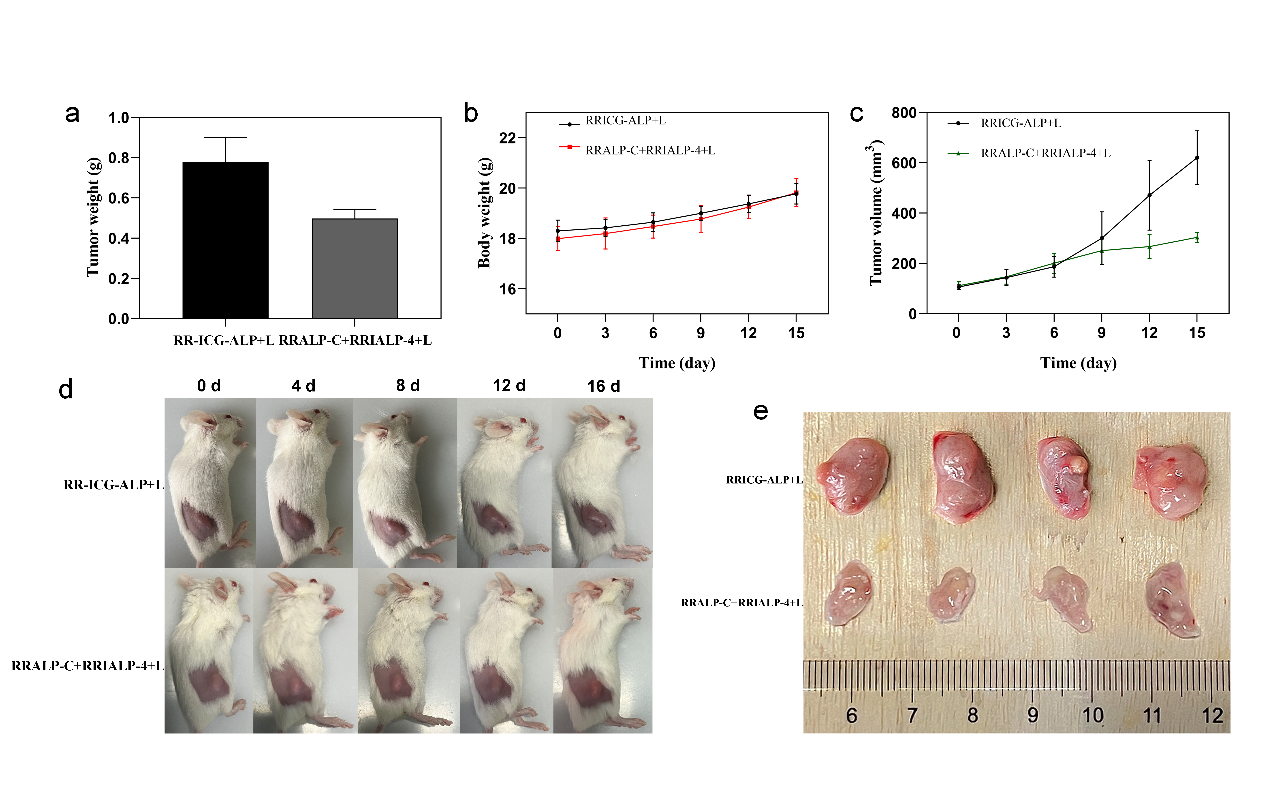


**Fig. S17.** In vivo synergistic therapy of RR-ICG-ALP and single liosome combi group (RRALP-C+RRIALP-4+L)


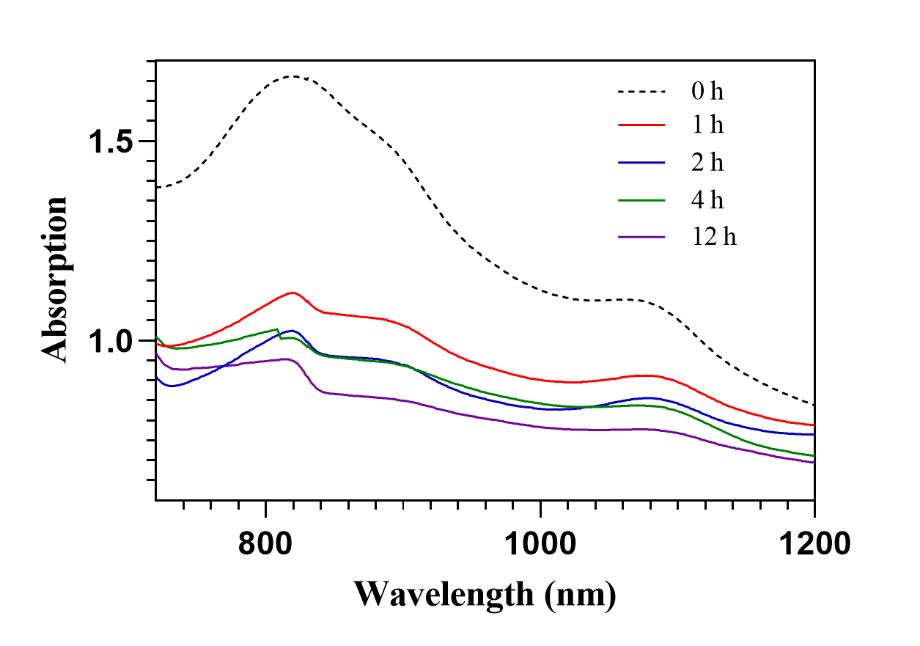


**Fig. S18.** Absorption peaks of purified cell membrane after Incubated with IALP-4.
